# Palliative radiotherapy in symptomatic pelvic soft tissue tumors (PallSoft)– protocol for a national, randomized, non-inferiority study

**DOI:** 10.1186/s12885-025-14424-1

**Published:** 2025-07-01

**Authors:** Kjersti Skipar, Maren S. Ørvik, Christoph Evers, Lise Balteskard, Christian Ekanger, Liv Ellen Giske, Kjersti Ødegaard, Elin H. Østrem, Cecilie S. Nordstrand, Hanne Tøndel, Carsten Nieder, Marianne G. Guren, Stein Kaasa, Harald B. Ragnum

**Affiliations:** 1 Department of Oncology, Telemark Hospital Trust, Ulefossvegen 55, 3710 Skien, Norway; 2 Center for Cancer Treatment, Hospital of Southern Norway, Kristiansand, Norway; 3 Department of Oncology, University Hospital of North Norway, Tromsø, Norway; 4 Department of Oncology and Medical Physics, Haukeland University Hospital, Bergen, Norway; 5 Department of Oncology, Gjøvik Hospital, Innlandet Hospital Trust, Gjøvik, Norway; 6 Department of Oncology, Stavanger University Hospital, Stavanger, Norway; 7 Section of Oncology, Drammen Hospital, Vestre Viken Hospital Trust, Drammen, Norway; 8 Department of Oncology, Ålesund Hospital, Møre and Romsdal Hospital Trust, Ålesund, Norway; 9 Cancer Clinic, St. Olavs Hospital Trust, Trondheim University Hospital, Trondheim, Norway; 10 Department of Oncology and Palliative Medicine, Nordland Hospital, Bodø, Norway; 11 Department of Clinical Medicine, Faculty of Health Sciences, University of Tromsø, Tromsø, Norway; 12 Department of Oncology, Oslo University Hospital, Oslo, Norway; 13 Institute of Clinical Medicine, University of Oslo, Oslo, Norway; 14 European Palliative Care Research Centre (PRC), Oslo, Norway

## Abstract

**Background:**

Palliative radiotherapy is essential in the management of patients with symptomatic pelvic soft tissue tumors, often providing rapid and efficient symptom relief. No standard treatment recommendations currently exist, yielding large differences in patient management across cancer types and institutions. PallSoft is a national, phase III, non-inferiority study aiming to compare two short-course radiotherapy approaches for these patients.

**Methods:**

200 patients will be recruited from 11 institutions over 2–4 years. Patients with either gastrointestinal, urological or gynecological cancers, referred to palliative radiotherapy due to a symptomatic pelvic soft tissue tumor, are eligible for study inclusion. Patients will define their target symptom and be randomly assigned to treatment with either 1 fraction of 8 Gy (Gy) (arm A) or 5 fractions of 5 Gy (arm B). An additional fraction of 8 Gy may be offered to patients in arm A if unsatisfactory symptomatic effect occurs, evaluated according to predefined criteria. The primary objective is to investigate whether the patient-reported target symptom relief in arm A is non-inferior to arm B, assessed on a Numeric Rating Scale (NRS). Secondary objectives are physician-assessed bowel and bladder toxicities and overall survival. Explorative objectives include evaluations of health-related quality of life, general patient satisfaction and health economic aspects. Prognostic models for survival prediction and predictive biomarkers for radiotherapy response will be explored. Statistical analyses using linear regression models and survival analyses will be employed.

**Discussion:**

We aim to provide evidence of the most optimal palliative radiotherapy regimen for patients with symptomatic pelvic soft tissue tumors, and thereby contribute to establish a standard-of care for these patients. The participation of all radiotherapy units in Norway may ease national implementation of study results.

**Trial registration:**

Registered at ClinicalTrials.gov (Palliative Radiotherapy in Symptomatic Pelvic Soft Tissue Tumors, NCT06398314) on May 3rd, 2024. First patient enrollment in February 2025. All hospitals are currently recruiting.

**Trial sponsor:**

Telemark Hospital Trust.

**Supplementary Information:**

The online version contains supplementary material available at 10.1186/s12885-025-14424-1.

## Background

Palliative radiotherapy is cost-effective and time-efficient, and provides local symptom alleviation in patients with metastatic cancer [1]. The importance and effectiveness of radiotherapy in the treatment of cancer patients are firmly established, however, radiotherapy research is less prioritized compared to research on medical oncology and precision oncology [2]. Moreover, radiotherapy studies often lack focus on health services research, palliative care and quality of life (QoL) [2]. Increased effort in palliative radiotherapy research, with emphasized focus on patient-centered and patient-reported outcomes, is required [2–4].

Pelvic soft tissue tumors, originating from gastrointestinal, urological or gynecological cancers, are often symptomatic in terms of pain, bleeding, or bowel/lower urinary tract/vaginal dysfunction, and symptoms may significantly impact patient QoL [5]. Clinical trials and clinical practice have demonstrated that palliative radiotherapy provides efficient symptom relief in patients with pelvic soft tissue tumors [1, 6–16]. However, previous studies are mainly retrospective and difficult to compare due to a variety of radiotherapy regimens applied. The chosen radiotherapy schedule therefore often depends on cancer diagnosis and institutional preference, and a standard of care needs to be established.

Palliative patients have a limited life expectancy, emphasizing the need for palliative radiotherapy to provide rapid symptom control within a limited overall treatment time. A short-course radiotherapy regimen with few radiotherapy fractions of a high radiation dose is therefore favorable. Moreover, in an evolving oncologic landscape with increasing systemic therapies, a short-course radiotherapy regimen minimizes the interruption from systemic treatment and is applicable when both local symptom relief and systemic disease control are warranted. Finally, a short-course radiotherapy regimen may offer improved exploitation of health care resources.

A short-course radiotherapy regimen of 1–2 fractions has proven to be equally effective and tolerable as prolonged regimens in palliative patients with either painful bone metastases or thoracic symptoms [17–23]. Further, in rectal cancer, a short-course radiotherapy regimen of five fractions has proven effective and tolerable in both elderly patients and patients with metastatic disease [24, 25]. However, for patients with symptomatic pelvic soft tissue tumors in general, the optimal radiotherapy regimen is yet to be established [6, 7, 13, 16].

Maximum symptom control after palliative radiotherapy is achieved in weeks to months, hence, patients with life expectancy limited to a few weeks would likely not benefit from treatment [26]. Clinicians tend to overestimate patient survival [26]. Prognostic models that may be helpful in survival prediction have been developed, but their applicability in patients receiving palliative radiotherapy for symptomatic pelvic soft tissue tumors remains to be validated [26–31]. Furthermore, predictive factors associated with radiotherapy resistance, such as tumor hypoxia and programmed cell death ligand 1 (PD-L1) expression, could provide useful information in clinical decision-making [32–36]. However, their predictive value in palliative radiotherapy is currently unclear.

The PallSoft study aims to address the current evidence gap regarding the most optimal radiotherapy regimen for palliative patients with symptomatic pelvic soft tissue tumors. The randomized and pragmatic study design, and the participation of all radiotherapy units in Norway, will provide data approximating a real-world situation, which in turn will enhance clinical applicability and facilitate implementation of study results.

## Methods/Design

PallSoft is a prospective, randomized, open-label, national, parallel-arm non-inferiority phase III study. All Norwegian hospitals with a radiotherapy unit is participating in the study (11 hospitals).

The study is approved by the Regional Committee for Medical and Health research in South-Eastern Norway and the data protection office at each participating center.

A signed informed consent will be obtained from all patients by dedicated study personnel. Study conductance will be in accordance with the World Medical Association Declaration of Helsinki, Council for International Organizations of Medical Sciences International Ethical Guidelines, ICH Good Clinical Practice Guidelines and General Data Protection Regulation.

### Intervention

Eligible patients will be randomized to receive palliative radiotherapy with either 1–2 fractions of 8 Gy (arm A) or 5 fractions of 5 Gy (arm B). A computer-generated random allocation ratio of 1:1 is applied, and central randomization is performed. Randomization will be stratified on the target symptoms pain and bleeding.

Patients in arm A, initially receiving one fraction of 8 Gy, *may* be offered a second fraction of 8 Gy if there is an unsatisfactory response after four weeks, determined by presence of at least one of the following criteria:

- Equal to or less than a two-point improvement in NRS score compared to baseline.
- Initial symptomatic effect, but rapid worsening of symptoms according to NRS score.

Total tumor doses in arm A and B, indicated in equivalent dose in 2 Gy fractions (EQD2), are 12/24 Gy, and 31.3 Gy, respectively, when an alpha beta ratio of 10 is applied [37]. The radiotherapy regimen of patients in arm B may be modified during the intervention (fractionation changes, intervention pauses) due to extensive toxicity, deterioration in the general condition, intercurrent disease, or poor compliance. If the intervention is paused, a maximum intervention length of 10 weekdays is allowed, otherwise radiotherapy will be recorded as incomplete. Both radiotherapy regimens are currently used in routine clinical practice for the same indications, and are experienced as effective and well tolerated. Treatment efficacy and toxicities will be closely monitored, and all adverse events (AE) ≥ grade 3 and all serious adverse events (SAE) will be registered until four weeks of follow-up. After four weeks of follow up, only AEs ≥ grad 3 and SAEs with possible, probable or definite causality with radiotherapy will be reported.

### Objectives

**Fig. 1:**
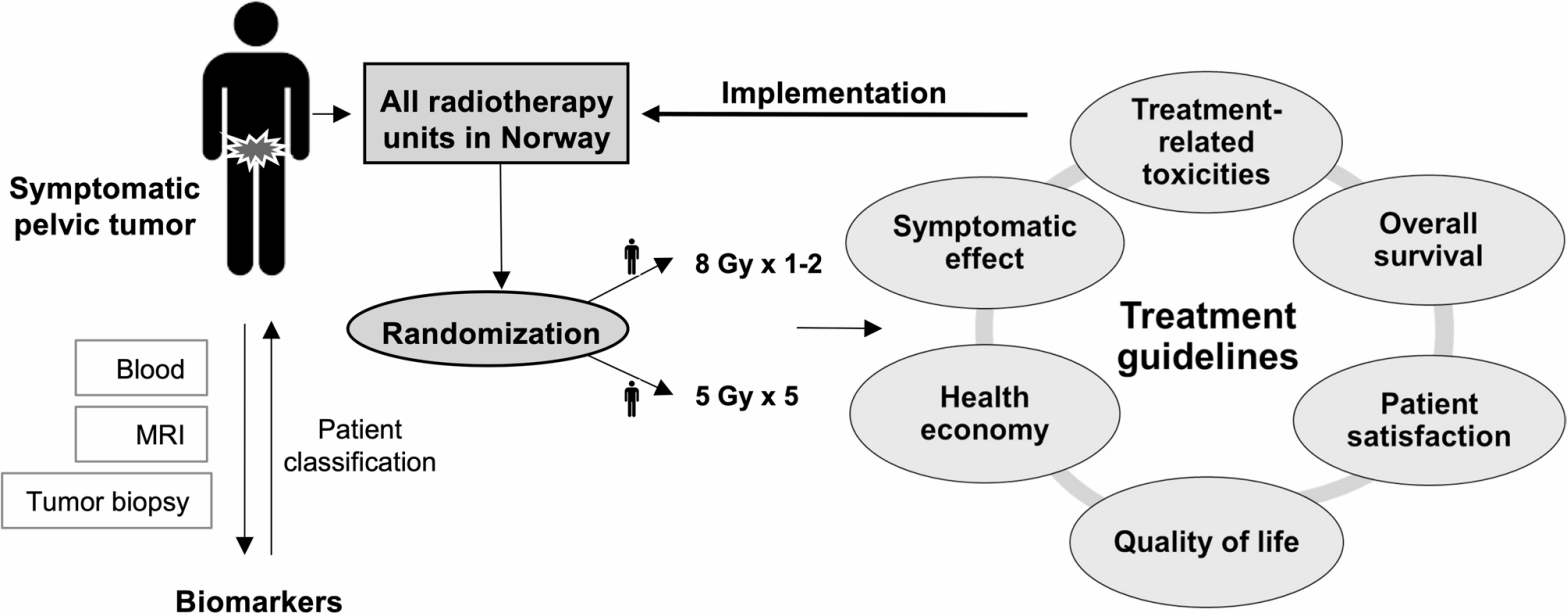
Schematic overview of study objectives. Patients will be randomized to receive a radiotherapy regimen with either 1–2 fractions of 8 Gray (Gy) or 5 fractions of 5 Gy. Outcome measures include symptomatic effect, treatment-related toxicities, overall survival, patient satisfaction, quality of life and health economy. Predictive and prognostic biomarkers will be assessed

Primary objective is to investigate whether a regimen with 1–2 fractions of 8 Gy (Gy) (arm A) is non-inferior to a regimen with 5 fractions of 5 Gy (arm B) with respect to patient-reported target symptom relief after 12 weeks of follow-up. Secondary objectives are to compare physician-reported bowel and bladder toxicities and overall survival (OS) between study arms. Exploratory objectives are to compare patient-reported outcome measures (QoL, symptom relief and overall patient satisfaction), all relevant physician-reported toxicities, and health resource utilization. In addition, prognostic models for patient classification and predictive biomarkers of radiotherapy response will be explored. An overview of study objectives is presented in Fig. 1.

### Inclusion criteria

- Histologically verified primary cancer originated from gastrointestinal, urological or gynecological organs (histological verification can be performed on any lesion).
- Patients unsuitable for curative treatment (advanced disease or medical contradictions [i.e. comorbidity, old age, poor general condition]).
- Primary, residual, recurrent or metastatic pelvic soft tissue tumor from the above-mentioned cancers not amenable for curative treatment.
- Tumor-related symptoms including pain, bleeding, bowel dysfunction, lower urinary tract dysfunction or vaginal dysfunction.
- Candidate for palliative radiotherapy according to both study arms.
- Patient-reported average severity of symptoms ≥ 4 on an NRS- scale of 0–10.
- ≥ 18 years of age.
- Speaks and understands Norwegian or English.
- Ability to understand and willing to sign a written informed consent.
- Eastern Cooperative Oncology Group (ECOG) performance status 0–3.
- Expected survival > 12 weeks.
- Able to pause systemic cancer treatment for one week prior to, during, and one week after radiotherapy (except antihormonal treatment).
- Women of childbearing potential (WOCBP) must have a negative highly sensitive serum pregnancy test within 72 h prior to randomization and agree to the use of highly effective birth control methods or abstain from sexual activity from randomization and until completed study intervention.

### Exclusion criteria

- Neuroendocrine histology.
- Sarcoma or sarcomatous components in the histology.
- Tumors that originate from bony metastases without a soft tissue component, or tumors where the soft tissue component constitutes of less than 50% of the total tumor volume or the soft tissue component is unlikely to contribute to the target symptom.
- Unable to complete study questionnaires.
- Ongoing treatment with an investigational drug.
- Planned inclusion in another interventional clinical study within 4 weeks after radiotherapy.
- Pregnancy (due to risk of teratogenic and abortifacient effects of radiotherapy).

### Evaluations

**Fig. 2:**
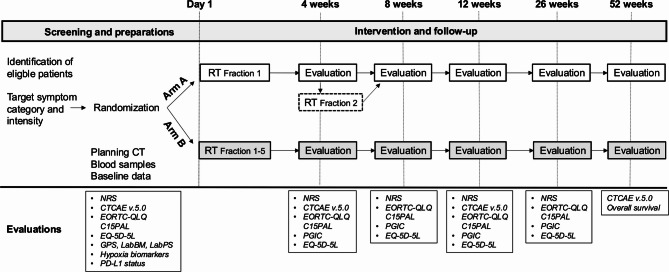
Study timeline with evaluations and corresponding time points. The second fraction of radiotherapy (RT) in arm A (*) is indicated with dotted lines. Prognostic scores, hypoxia biomarkers and PD-L1- status will be assessed on blood tests, MRI and/or tumor biopsies available from standard patient diagnostic work-up, treatment and/or follow-up

Scheduled evaluations and corresponding timepoints are provided in Fig. 2.

#### Baseline clinical data

Baseline clinical variables to be registered is presented in Table 1.

**Table 1:** Baseline clinical variables

• Age
• Sex
• Smoking status
• ECOG performance status (PS)
• Cancer diagnosis and extension (primary origin only/locoregional disease below common iliac artery/locoregional disease below aortic bifurcation/metastatic)
• Histology
• Relevant comorbidity
• Concomitant supporting medications (analgesics, antifibrinolytic agents, anticoagulants, laxatives, antidiarrheal agents, steroids, antiemetics)
• Systemic cancer treatment within last 6 weeks
• Prior radiotherapy with overlapping field(s)
• Date of previous Magnetic Resonance Imaging (MRI)
• Date of previous tumor biopsies
• Baseline blood tests performed as part of routine follow-up and preparation for radiotherapy, (hemoglobin [g/dL], platelets [10^9^/L], albumin [g/L], CRP [mg/L] and LDH [U/L]

#### Patient-reported outcome measures (PROMs)

Patients must define a target symptom, denoted as the patients‘ main complaint, within the five predefined categories pain, bleeding, bowel dysfunction, lower urinary tract dysfunction, or vaginal dysfunction. Other symptoms (maximum two), related to the pelvic soft tissue tumor planned irradiated, may also be assessed separately.

The following measurements will be performed.

- Intensity of target symptom, and, when present, of additional symptoms, assessed by a Numeric Rating Scale (NRS) of 1–10.
- QoL (The European Organization for Research and Treatment of Cancer Quality of Life Questionnaire - Core15-PALliative [EORTC QLQ-C15 PAL] and EuroQol-5 Dimensions-5 Levels [EQ-5D-5 L].
- Patient global impression of change (PGIC).

#### Toxicities

Physician-reported toxicities will be assessed according to Common Terminology Criteria for Adverse Events (CTCAE) version 5.0.

#### Prognostic models and predictive biomarkers

Prognostic models including the Glasgow prognostic score (GPS), the LabBM score, and the LabPS-score will be assessed at baseline [27, 28, 30, 31] (Table 2). Predictive biomarkers will be assessed in relevant patients where multiparametric (mp) MRI and/or tumor biopsies are available from diagnostic work-up and treatment. Available imaging and biopsies from the planned GTV will be prioritized for analyses. Hypoxia biomarkers will be assessed on mpMRI using the Consumption and Supply-based Hypoxia (CSH)-imaging method [33, 38], and on tumor biopsies using molecular analyses to explore expression of hypoxia-activated proteins and genes [32, 39, 40]. PD-L1 expression will be assessed by immunohistochemical staining with calculations of Combined Positive Score (CPS) and Total Proportion Score (TPS) [36, 41].

**Table 2:** Prognostic models

	Variables
GPS	CRP, albumin
LabBM	CRP, albumin, LDH, hemoglobin, platelets
LabPS	CRP, albumin, LDH, hemoglobin, platelets, ECOG PS

#### Radiotherapy reporting

Radiotherapy planning parameters (target volume, organs at risk [OAR]) are defined in the study protocol, while other procedures for radiotherapy planning and conduction will be in accordance with institutional practice on each participating hospital. An overview of planning volumes and corresponding doses to be reported in the study is presented in Table 3. Bowel bag refers to contouring of the entire intraperitoneal space and not the specific bowel loops.

**Table 3:** Radiotherapy planning volumes and doses

	**Volume** (cm^3^)	**Definition**	**Dose to be reported** (Gy)
Target	GTV	Gross Tumor Volume	-
	CTV	Clinical Target Volume	CTV D98
	PTV	Planning Target Volume	PTV D95 PTV D_min (0.003cm_^3^_/optional)_ PTV D_max (0.003cm_^3^_/optional)_
OAR	Bladder	Whole organ	D_max (0.1 cm_^3^_)_ Mean dose
	Rectum	From top of anal canal to the rectosigmoid transition	D_max (0.1 cm_^3^_)_ Mean dose
	Anal canal	3 cm from anus in axial plane	D_max (0.1 cm_^3^_)_ Mean dose
	Partial bowel bag	From 2 cm above upper limit of PTV to 2 cm below the lower limit of PTV in axial plane	D_max (0.1 cm_^3^_)_ Mean dose

Data on radiotherapy technique, matching structure(s) for control of positioning, and procedures of bladder filling will also be collected. If a second radiotherapy fraction is offered to patients in arm A, a Cone Beam Computed Tomography (CBCT), alternatively kV/kV when suitable, is recommended for control of positioning. The treating physician/oncologist will determine whether replanning of radiotherapy is necessary. Reirradiation within the same planning target volume (PTV) after the four week follow-up will be reported at each follow-up visit.

#### Health economics

Differences in health service costs related to attendance at the radiotherapy unit, transportation and hospital submissions will be evaluated through registration of costs from medical records.

### Statistical analysis

#### Sample size determination

The sample size determination is based on a one-sided two-sample *t*-test, with the following assumptions:

- 2.5% significance level (equivalent to a two-sided 95% confidence interval).
- Standard deviation (SD) of the primary endpoint equal to 2.
- Non-inferiority margin (NIM) of 1.

With an expected exclusion from intention to treat (ITT) - population of 25% [8, 9], a total number of 200 patients will be randomized to reach a power of 85%. This number of patients also provide sufficient power to claim non-inferiority of patient-reported target symptom relief at 4 and 8 weeks.

#### Choice of non-inferiority margin

The non-inferiority margin of the difference in primary endpoint between the two study arms is set to 1 point, which is 50% of the considered minimum clinically important difference based on clinical trials investigating cancer pain [42, 43]. The same non-inferiority margin is currently applied in an ongoing clinical non-inferiority trial (PARASTOP) investigating cancer pain (ClinicalTrials.gov identifier NCT05051735). To our judgement, given a mean difference in target symptom relief of less than 1 point, radiotherapy with 1–2 fractions of 8 Gy is preferred over 5 fractions of 5 Gy.

#### Data analyses

Baseline data will be presented with descriptive statistics; continuous variables as means with standard deviations and categorical variables as frequencies with percentages. Differences between study arms will be denoted as statistically significant if the P values are less than the predefined significance level of 5%. Correspondingly, a confidence level of 95% will be used for the two-sided confidence interval (CI) in linear regression models.

Data analyses for primary and secondary endpoints are defined in Table 4.

**Table 4:** Planned analyses for primary and secondary objectives

**Primary research question**	**Data analyses**
Is radiotherapy in arm A non-inferior to arm B with respect to patient-reported target symptom relief? *Endpoint*: Change in average (last 24 h) target symptom intensity from baseline after 12 weeks follow-up *Assessment*: NRS score (0–10)	Linear regression models for estimate of the difference between study arms. Non-inferiority will be claimed if the lower bound of the two-sided 95% confidence interval is above the non-inferiority margin of -1 (negative values indicate worse outcome for arm A). Statistical tests for interaction in multivariable linear regression model will be used to assess heterogeneity of radiotherapy response across different target symptoms. Sensitivity analyses will be performed to account for patient drop-out.

**Secondary research questions**	**Data analyses**
Is the efficacy of radiotherapy in arm A comparable to arm B with respect to: 1. *Physician-reported toxicities?* *Endpoint*: Bladder and bowel toxicity (4,12 and 52 weeks). *Assessment*: CTCAE version 5.0. 2. *Overall survival?* *Endpoint*: Overall survival (OS) *Assessment*: End of study	1. Data will be reported as means, 95% confidence intervals, medians or frequencies as appropriate. 2. Kaplan Meier-curves will be compared with the log-rank test and univariable and multivariable Cox proportional-hazards regression will be applied.

Data analyses of exploratory endpoints will be regarded as hypothesis-generating, post-hoc analyses. Continuous endpoints will typically be analyzed by linear regression or median regression, as appropriate. Dichotomous endpoints will typically be analyzed by logistic regression.

Statistical analyses will be performed using STATA v18 (Stata Corporation LP; College Station, TX, USA).

### Organizational issues

A Trial Steering Committee will oversee the conduction of the study. An independent Data Monitoring Committee that includes an independent statistician and two independent clinicians with experience in palliative radiotherapy, will evaluate the safety of study intervention after the inclusion of 70 patients. Due to clinical experience with the two radiotherapy regimens applied, the safety concerns are small, and study inclusion will continue while awaiting the safety evaluation.

The study is organized with a project leader group that includes the project leader, the national coordinating investigator, two principal investigators with extensive experience in palliative radiotherapy and a scientific advisor with significant experience in palliative care and management of clinical studies. In addition, the study is organized with a study group, including all principal investigators (MDs), one medical physicist, one radiation therapist and the study coordinator.

The study will be administered from the coordinating center, including development and administration of Case Report Forms (CRFs), monitoring of data quality and preparation of the final study report. Results will be published in peer-reviewed journals with authorship in adherence with the Vancouver rules.

## Discussion

Increased effort in palliative radiotherapy research, emphasizing patient-centered and patient-reported outcomes, is required. Despite extensive clinical experience regarding the efficacy of palliative radiotherapy in patients with symptomatic pelvic soft tissue tumors, no consensus on patient management exists, rendering large differences in patient management across cancer types and institutions. Patients with symptomatic pelvic soft tissue tumors include large cancer groups such as prostate, gastrointestinal and gynecologic cancers. Evidence of the most optimal radiotherapy regimen would therefore benefit many future cancer patients. Moreover, as available health care resources are challenged by the increasing cancer incidence and prevalence [44], improved exploitation of health care resources is required. Positive results from this study may contribute to that aspect.

To comprehend the large variations in radiotherapy schedules applied for the current indication today, a pragmatic and national approach was considered important to enable clinical impact. However, challenges with this approach were evident. Treatment practices across institutions and diagnoses were required to unite in a common, randomized design that also preserved the ethical aspects of keeping both patient burden and potential risks at a minimum level. For the latter, an optional, non-randomized radiotherapy fraction in arm A was considered necessary if suboptimal symptomatic effect occurred. Hence, non-inferiority can only be established for a regimen of up to two fractions.

Albeit uncertainties related to inclusion of a heterogenous study population, it was considered appropriate to evaluate patients with symptomatic pelvic soft tissue tumors as an entity, due to the similar management of these patients across diagnoses and symptoms. Moreover, the total doses in EQD2 of 12–24 Gy in arm A and 31.3 Gy in arm B, represent a dose range transferable to other regimens such as 10 fractions of 3 Gy (EQD2 of 32.5 Gy) or 5 fractions of 4 Gy (EQD2 of 23.3 Gy), which are commonly applied regimens across diagnoses. It should be noted that different tumor sites have different alpha/beta ratios, and, hence, different fractionation sensitivities [45]. Nevertheless, the fractionation sensitivity of tumor cells may be less relevant for therapeutic effect to palliative radiotherapy, as illustrated by the importance of endothelial damage for control of tumor related bleeding [46].

Ultimately, the pragmatic study design will provide data approximating a real-world situation, which may facilitate clinical implementation of study results. The focus on patient-centered and patient-reported endpoints will provide systematic and warranted data on important aspects in assessment of treatment benefit, including QoL.

The rationale for a non-inferiority design is based on the assumption that 1–2 fractions of 8 Gy will be the preferred treatment regimen if non-inferior symptomatic effect is established, and toxicities and overall survival are comparable. This regimen imposes less burden on patients and health care resources, and a superiority study was considered unnecessary for implementation of study results. Although superiority studies that fail to reject the null hypothesis are frequently interpreted as showing no differences between groups, they could be interpreted as indeterminate [43].

The primary endpoint will be assessed by NRS score, where current validation is limited to the evaluation of pain [47, 48]. To our knowledge, no single, validated tool for assessment across different symptoms exists. Based on previous studies, the majority of included patients is expected to present with either pain or bleeding as their target symptom [8–10, 16]. To provide partial validation of NRS in the assessment of bleeding severity, a simultaneous registration of physician-assessed CTCAE-grading will be performed. Based on the current evidence and clinical experience regarding the excellent symptomatic effect of palliative radiotherapy on tumor bleeding [8, 9, 13, 46, 49], randomization will be stratified to ensure equal distribution of target symptoms bleeding and pain between study arms.

Development and continuous validation of prognostic models for patient classification will increase knowledge about when to offer and when to abstain from palliative radiotherapy. Moreover, predictive biomarkers might provide enhanced insight of the radiotherapy response, with potential value in patient management. However, the clinical relevance of predictive biomarkers in palliative radiotherapy is currently unexplored, and evident ethical issues arise when introducing additional, invasive procedures in palliative patients solely for experimental purposes. Hence, the PallSoft study will explore predictive biomarkers derived from medical imaging and biopsies that are already acquired in standard diagnostics and treatment. Albeit with great uncertainty, this hypothesis-generating method might bridge further studies and substantiate a prospective future approach.

## Electronic supplementary material

Below is the link to the electronic supplementary material.

Supplementary Material 1

Supplementary Material 2

Supplementary Material 3

## Data Availability

No datasets have been generated or analysed during the current study.

## Abbreviations

AE: Adverse event
CBCT: Cone beam computed tomography
CPS: Combined positive score
CRF: Case report form
CRP: C-reactive protein
CSH-imaging: Consumption- and supply based hypoxia imaging
CTCAE: Common terminology criteria for adverse events
ECOG: Eastern cooperative oncology group
EORTC QLQ-C15 PAL: The European organization for research and treatment of cancer quality of life questionnaire - core15-PALliative
EQ-5D-5L: EuroQol-5 dimensions-5 levels
EQD2: 2 Gy equivalent dose
GTV: Gross tumor volume
Gy: Gray
ITT: Intention to treat
kV: Kilovoltage
LDH: Lactate dehydrogenase
mp: Multiparametric
MRI: Magnetic resonance imaging
NIM: Non-inferiority margin
NRS: Numeric rating scale
OAR: Organ at risk
OS: Overall survival
QoL: Quality of life
PD-L1: Programmed death- ligand 1
PGIC: Patient global impression of change
PROM: Patient-reported outcome measures
SAE: Serious adverse event
TPS: Total proportion score
WOCBP: Women of childbearing potential
