# Supplementary material for: Palliative radiotherapy in symptomatic pelvic soft tissue tumors (PallSoft)– protocol for a national, randomized, non-inferiority study
Source: BMC Cancer. 2025 Jul 1;25:1051. doi: 10.1186/s12885-025-14424-1 (PMC12211963; doi:10.1186/s12885-025-14424-1)
Supplement: Supplementary file 2 — Supplementary Material 2 [file 12885_2025_14424_MOESM2_ESM.pdf]

PallSoft- PALLiative radiotherapy in symptomatic pelvic SOFT tissue tumors

A randomized, open-label, multicenter, national, parallel-arm non-inferiority phase III trial investigating whether palliative radiotherapy delivered in 1-2 fractions is non-inferior to palliative radiotherapy delivered in 5 fractions with respect to change in target symptom intensity in patients with pelvic soft tissue tumors.

Protocol overview

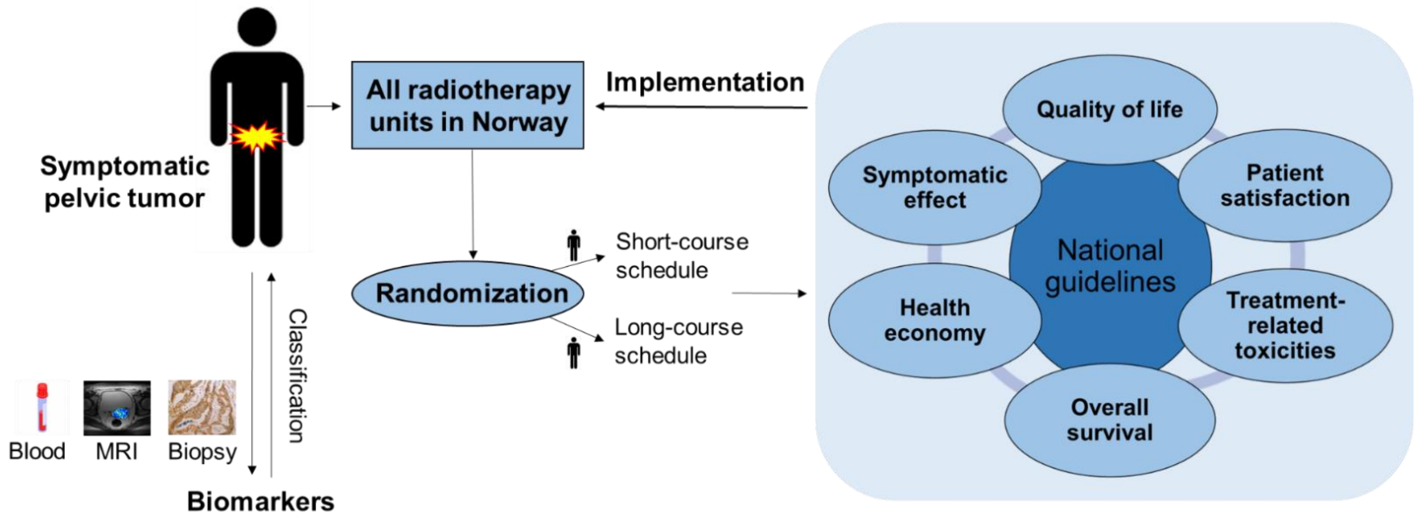

Number of arms:

Two

Masking:

No masking

Primary Health Measurement:

PROM (Patient Reported Outcome Measures)

Disease:

Gastrointestinal, urological or gynecological cancer

Protocol Number:

September 2024- version 2.3.2

Brief Title:

PALLSOFT- Palliative radiotherapy for pelvic soft tissue tumors

Study phase:

III

Acronym:

PallSoft

Sponsor Name:

Telemark Hospital Trust

Contact Address: Ulefossvegen 55, 3710 Skien, Norway

Regulatory Agency Identifier Number(s)

REK 606316

Approval Date:

November 22, 2023

Medical Monitor Name and Contact Information:

Will be provided separately

Sponsor Signatory:

Irene Larsen Sørbel 15.05.2025

Irene Larsen Sørbel **Date**

Head of Division of Medical services, Telemark Hospital Trust

Lokal PI Signatory:

Name, department and hospital **Date**

Protocol Amendment Summary of Changes Table

Version	Date	Section updated	Summary of changes	Reason for change
1.0	21.03.2023		NA	Initial protocol
2.0	13.10.2023	Contact details<br><br><br>3<br>5<br>5.1<br>6.8 and 8.1<br>2.1.3, 2.2.2.1, 2.3, 8.4.2, 8.8<br>2, 8.2.1	New head of department (sponsor)<br>New project leader<br>Project management group added<br>Clarification objectives/endpoints<br>Updated recruitment procedure<br>Updated inclusion criteria<br>Updated concomitant medications<br>Added exploratory endpoint (hypoxia biomarkers)<br>Narrative clarifications	Feedback from Regional Committee for Medical Research Ethics South East Norway (REC South East)<br><br>Discussions in the study group
2.1	.06.02.2024	5<br>8.1<br>8.4.2<br>8.2.1.1	Updated recruitment procedures<br>Updated baseline status<br>Updated planned procedures<br>Elaborative information	Feedback from REC South East<br><br>Discussions in the study group
2.2	April/May 2024	9.5, 9.6<br><br>1.1, 2.1.3, 2.2.2.1, 3, 8.4.2<br><br>5	Statistical analyses updated/elaborated<br>Fig.1 updated according to SoA<br>Exploratory endpoints updated<br>Title page updated<br>Narrative clarifications<br>Recruitment procedure updated	Discussions with study statistician<br>Discussions in study group<br><br>Feedback from REC South East
2.3.1	12.07.2024	5	Recruitment procedure updated	Feedback from REC South East

		1.2<br>1.2, 2.3.1, 3, 4.1, 8.2.1, 4.1<br>6.3<br>1.2, 5.1, 8.3.5,	SoA clarified<br>Assessment PROM at 6 months added. Fig.1 clarified<br>Updated randomization stratification<br>WOCBP updated	Discussion in study group
2.3.2		8.2.1<br>1.2, 6.2	NRS baseline as demographic variable<br>Narrative clarifications<br>Updated Contact Details<br>Fig.1 updated (clarifications)	Discussion in study group
2.4		5.1<br>1.2, 2.1, 3, 4.1, Fig.1, 8.2<br>6.2, 8.1, 8.5.1	Inclusion criterium added<br>PROMS at 8 weeks added<br>Narrative clarifications	Discussion in study group and DMC

Contact Details:

**Sponsor:**	Name and title:
Telemark Hospital Trust	Irene Larsen Sørbel, Head of Division of Medical services
	Telemark Hospital Trust
	E-mail: Irene.Larsen.Sorbel@sthf.no
	**Project leader, Project management group**
	Name and title: Harald Bull Ragnum, MD, PhD
	Department of oncology, Telemark Hospital Trust
	Tel: +47 907 92 571
	E-mail: harrag@sthf.no
	**National Coordinating Investigator, Project management group**
	Name and title: Kjersti Skipar, MD
	Department of oncology, Telemark Hospital Trust
	Tel: +47 984 44 114
	E-mail: kjeski@sthf.no
**Participating Site:** Nordland Hospital	**Principal investigator (PI), Project management group**
	Name and title: Carsten Nieder¸MD, prof.
	Department of oncology, Nordland Hospital
	Tel: +47 481 27 634
	E-mail: Carsten.Nieder@nordlandssykehuset.no
**Participating Site**:	**Principal investigator (PI)**
Møre and Romsdal Hospital Trust	Name and title: Cecilie Soma Nordstrand, MD
	Department of oncology, Møre and Romsdal Hospital Trust
	Tel: +47 97191463
	E-mail: Cecilie.Soma.Nordstrand@helse-mr.no

**Participating Site**:	**Principal investigator (PI)**
Stavanger University Hospital	Name and title: Kjersti Ødegaard, MD<br>Department of Oncology, Stavanger University Hospital HF<br>Tel: +47 51 51 89 06/+47 412 19 892<br>E-mail: odkj@sus.no
**Participating Site**:	**Principal investigator (PI)**
Hospital of Southern Norway	Name and title: Christoph Evers, MD<br>Address: Department of Oncology, Hospital of Southern Norway<br>Tel: +47 461 24 398<br>E-mail: Christoph.Evers@sshf.no
**Participating Site**:	**Principal investigator (PI), Project management group**
Oslo University Hospital	Name and title: Marianne G. Guren, MD, prof<br>Department of Oncology, Oslo University Hospital<br>Tel: +47 997 13 594<br>E-mail: marianne.gronlie.guren@ous-hf.no<br>**Project management group**<br>Name and title: Stein Kaasa, MD, prof.<br>Department of Oncology, Oslo University Hospital<br>Tel: +47 918 97 001<br>E-mail: stein.kaasa@medisin.uo.no
**Participating Site**:	**Principal investigator (PI)**
Vestre Viken Hospital Trust	Name and title: Elin Østrem, MD<br>Department of Oncology, Vestre Viken Hospital Trust<br>Tel: +47 951 11 917<br>E-mail:Elin.Hoie.Ostrem@vestreviken.no
**Participating Site**:	**Principal investigator (PI)**
Innlandet Hospital Trust	Liv Ellen Giske, MD<br>Department of Oncology, Innlandet Hospital Trust

Tel: +47 905 36 624

E-mail: Liv.Ellen.Giske@sykehuset-innlandet.no

**Participating Site**:	**Principal investigator (PI)**
St.Olavs Hospital, Trondheim University Hospital	Hanne Tøndel, MD, PhD<br>Department of Oncology, St.Olavs Hospital, Trondheim University Hospital<br>Tel: +47 72 82 55 07<br>E-mail: Hanne.Tondel@stolav.no
**Participating Site**:	**Principal investigator (PI)**
Haukeland University Hospital	Christian Ekanger, MD<br>Department of Oncology, Haukeland University Hospital<br>Tel: +47 997 35 646<br>E-mail: christian.ekanger@helse-bergen.no
**Participating Site**:	**Principal investigator (PI)**
University Hospital of North-Norway	Lise Balteskard, MD, PhD<br>Department of Oncology, University Hospital of North-Norway<br>Tel: 412 45 027<br>E-mail: Lise.Balteskard@unn.no

Table of Content

Protocol Amendment Summary of Changes Table 4
Contact Details: 6
Table of Content 9
1. Protocol Summary 12
1.1. Synopsis 12
1.2. Schedule of Activities (SoA) 17
2. Introduction 20
2.1. Study Rationale 21
2.1.1. Principal Research Question 21
2.1.2. Secondary Research Questions 21
2.1.3. Exploratory Research Questions 21
2.1.4. Rationale for Timing of Endpoint Assessment 22
2.2. Background 22
2.2.1. Background Disease 22
2.2.2. Background Treatment 24
2.3. Benefit/Risk Assessment 28
3. Objectives, Endpoints and Assessments 30
4. Study Design 32
4.1. Overall Design 32
4.2. Scientific Rationale for Study Design 33
4.2.1. Participant Input into Design 33
4.3. Justification for Radiotherapy Dose 33
4.3.1. Second Radiotherapy Fraction in Arm A 33
4.4. End of Study Definition 34
5. Study Population 35
5.1. Inclusion Criteria 35
5.2. Exclusion Criteria 36
5.3. Lifestyle Considerations 37
5.4. Screen Failures 37
5.5. Criteria for Temporarily Delaying 37
6. Study Intervention(s) and Concomitant Therapy 38
6.1. Study Intervention 38
6.2. Radiotherapy planning 38
6.3. Measures to Minimize Bias: Randomization and Blinding 39
6.4. Study Intervention Compliance 40
6.5. Radiotherapy Treatment Course Modification 40
6.6. Continued Access to Study Intervention after the End of the Study 40

6.7. Treatment of Overdose 40
6.8. Concomitant Therapy 41

7. Discontinuation of Study Intervention and Participant Discontinuation/Withdrawal....42
7.1. Discontinuation of Study Intervention....42
7.1.1. Temporary Discontinuation 42
7.1.2. Rechallenge....42
7.2. Participant Discontinuation/Withdrawal from the Study....42
7.3. Lost to Follow-up....42

8. Study Assessments and Procedures....44
8.1. Baseline Demography and Current Medical History....45
8.2. Efficacy Assessments 46
8.2.1. Patient Reported Outcome Measures....46
8.2.2. Overall Survival....47
8.2.3. Health economics....47
8.3. Safety Assessments....47
8.3.1. Follow-up after 1 week....47
8.3.2. Physician-assessed toxicity CTCAE....47
8.3.3. ECOG Performance Status 48
8.3.4. Clinical Safety Laboratory Assessments 48
8.3.5. Pregnancy Testing....48
8.4. Other Assessments....48
8.4.1. Baseline Prognostic Scores....48
8.4.2. Predictive Biomarkers....49
8.5. Adverse Events (AEs), Serious Adverse Events (SAEs), and Other Safety Reporting....49
8.5.1. Time Period and Frequency of Collecting AE and SAE Information 50
8.5.2. Method of Detecting AEs and SAEs 50
8.5.3. Follow-up of AEs and SAEs....50
8.5.4. Regulatory Reporting Requirements for SAEs....51
8.5.5. Pregnancy....51
8.5.6. Disease-Related Events and/or Disease-Related Outcomes Not Qualifying as AEs or SAEs....51
8.6. Pharmacokinetics....52
8.7. Genetics and/or Pharmacogenomics....52
8.8. Biomarkers....52
8.9. Immunogenicity Assessments....52
8.10. Health Economics OR Medical Resource Utilization and Health Economics....52

9. Statistical Considerations....53
9.1. Choice of Non-inferiority Margin....53
9.2. Statistical Hypotheses....53
9.3. Sample size determination....53

9.4. Analysis Sets 54
9.5. Statistical analyses 55
9.5.1. General Considerations 55
9.5.2. Primary Endpoint 55
9.5.3. Secondary Endpoint 55
9.5.4. Exploratory endpoints 56
9.5.5. Safety Analysis 56
9.6. Interim Analysis 56

10. Supporting Documentation and Operational Considerations 57
10.1. Appendix 1: Regulatory, Ethical, and Study Oversight Considerations 57
10.1.1. Regulatory and Ethical Considerations 57
10.1.2. Informed Consent Process 57
10.1.3. Data Protection 58
10.1.4. Committees Structure 58
10.1.5. Data Quality Assurance 58
10.1.6. Source Documents 59
10.1.7. Study and Site Start and Closure 60
10.1.8. Publication Policy 61
10.2. Appendix 2: Clinical Laboratory Tests 62
10.3. Appendix 3: AEs and SAEs: Definitions and Procedures for Recording, Evaluating, Follow-up, and Reporting 63
10.3.1. Definition of AE 63
10.3.2. Definition of SAE 64
10.3.3. Recording and Follow-Up of AE and/or SAE 65
10.3.4. Reporting of SAEs 67
10.4. Appendix 4: Contraceptive and Barrier Guidance 69
10.5. Appendix 5: Abbreviations 70
10.6. Appendix 6: Common Terminology Criteria for Adverse Events V 5.0 72

11. References 73

1. Protocol Summary

1.1. Synopsis

Protocol Title:

PallSoft- Palliative radiotherapy in symptomatic pelvic soft tissue tumors

A randomized, open-label, national, parallel-arm non-inferiority phase III trial investigating whether palliative radiotherapy delivered in 1-2 fractions is non-inferior to palliative radiotherapy delivered in 5 fractions with respect to change in target symptom intensity in patients with pelvic soft tissue tumors.

Background:

Studies and clinical practice have proven palliative radiotherapy to provide efficient symptom relief in patients with symptomatic pelvic soft tissue tumors. However, studies are mainly retrospective and difficult to compare due to a variety of radiotherapy fractionation schedules used, and they lack data on patient-reported quality of life (QoL). Consequently, no recommended standard of care is currently established in Norway. Several regimen are employed with variations in both number of fractions and total radiation dose (indicated in gray [Gy]).

In 2009–2015, the prospective, phase II, non-randomized PallRad study was conducted at several radiotherapy centers in Norway, and investigated the patient-reported symptomatic effect of palliative radiotherapy in participants with rectal and prostate cancer. The study proved radiotherapy to provide efficient symptom palliation, and displayed feasibility in addressing this particular topic in a multicenter setting.

A randomized trial designed to provide evidence of the optimal radiotherapy schedule in patients with symptomatic pelvic soft tissue tumors is highly warranted. Given the limited life expectancy of palliative patients, a short-course radiotherapy schedule would be preferable provided efficient symptom relief and good health-related quality of life.

Study rationale:

The main objective is to assess whether 1–2 fractions of 8 Gy can provide non-inferior patient-reported target symptom relief compared to 5 fractions of 5 Gy in patients with symptomatic pelvic soft tissue tumors. The target symptom is defined as one of the following five symptoms related to the irradiated tumor: pain, bleeding, bowel dysfunction, lower urinary tract dysfunction, and vaginal dysfunction

Objectives, Endpoints, and Assessments:

Primary objective

Assess whether 1-2 fractions of 8 Gy is non-inferior to 5 fractions of 5 Gy with respect to target symptom relief.

Endpoint: Change in the average (last 24 hours) patient-reported target symptom intensity from baseline assessed after 12 weeks of follow -up

Assessment: Numerical Rating Scale (NRS) score (0-10)

Secondary objectives

To assess the efficacy of 1-2 fractions of 8 Gy compared to 5 fractions of 5 Gy with respect to:

- Physician-reported toxicities

  Endpoint: Bladder and bowel toxicity after 4, 12 and 52 weeks of follow-up

  Assessment: Common Terminology Criteria for Adverse Events (CTCAE) v 5.0

- Survival

  Endpoint: Overall survival (OS) assessed at the end of study

Exploratory objectives

1. *To assess the efficacy of 1-2 fractions of 8 Gy compared to 5 fractions of 5 Gy with respect to:*

- Patient-reported QoL

  Endpoint: Change in QoL from BL after 4, 8, 12 and 26 weeks of follow-up

  Assessment: European Organization for the Research and Treatment of Cancer Quality of Life Questionnaire-Core15 Palliative (EORTC QLQ-C15 PAL)

- Physician-reported toxicities

  Endpoint: All relevant toxicities after 4, 12 and 52 weeks of follow-up

  Assessment: CTCAE v 5.0

- Patient reported symptomatic effect

  Endpoints: Change in target symptom intensity from baseline after 4, 8 and 26 weeks of follow-up, and changes in intensity of symptoms related to the irradiated tumor other than target symptom (within the five predefined categories) from baseline after 4, 8, 12 and 26 weeks of follow-up

  Assessment: NRS score 0-10

- Patient satisfaction

  Endpoint: Assessment of patient satisfaction after 4, 8, 12 and 26 weeks of follow-up

  Assessment: Patient Global Impression of Change (PGIC)

2. To explore prognostic models for patient classification

Endpoint: Correlation between prognostic scores assessed at baseline and overall survival

Assessment: Glasgow Prognostic Score (GPS), LabPS- score and LabBM- score

3. To explore clinical factors as explanatory response variables

Endpoint: Correlation between clinical factors and symptomatic response

Assessment: Baseline registration of clinical factors from medical records

4. Assess hypoxia biomarkers on medical images and biopsies that are previously acquired for diagnosis or treatment, and explore their potential as explanatory response variables in individual patients.

Endpoint: Correlation between hypoxia biomarkers and symptomatic response

Assessment: Hypoxic fraction assessed by Consumption and Supply-based Hypoxia (CSH)-imaging on MRI (Magnetic Resonance Imaging) and hypoxia-activated protein and gene expression assessed by molecular analyses on tumor biopsies

5. Assess PD-L1 (Programmed Death-Ligand1)-status in biopsies previously acquired for diagnosis or treatment, and explore potential as explanatory response variable.

Endpoint: Correlation between PD-L1-status and symptomatic response

Assessment: PD-L1 expression level (all cells) assessed by immunohistochemistry

6. Compare the health-economic aspects in the two study arms regarding

- *Patient-reported QoL*

  Endpoint: Change in QoL compared to baseline assessed after 4, 8, 12 and 26 weeks

  Assessment: EQ-5D-5L (5 Level-EuroQol-5 Dimensions)

- *Health service costs*
  Endpoint: Difference in costs related to attendance at the radiotherapy unit, transportation and hospital submissions

  Assessment: Registration of costs from medical records

Overall Design:

This prospective, randomized, open-label, national, parallel-arm non-inferiority phase III study is designed to establish non-inferiority of radiotherapy with 1-2 fractions of 8 Gy to 5 fractions of 5 Gy with respect to patient-reported symptomatic relief. Patients in arm A will initially receive a single fraction of 8 Gy. If unsatisfactory symptomatic response after 4 weeks of follow-up, a

second fraction *may* be offered if at least one of the following two predefined criteria are met: 1) less than a 2 point improvement on the NRS-scale and/or 2) initial symptomatic effect, but rapid deterioration of symptoms, see Section 4.3.1). Patients in arm B will receive five consecutive fractions of 5 Gy. The study population includes patients with gastrointestinal, urological or gynecological cancers considered candidates for palliative radiotherapy, and with expected survival over 12 weeks. Eligible patients identified in the screening period will be randomized to either arm A or arm B with an allocation ratio of 1:1 after collection of a signed informed consent. Assessment of endpoints will be performed at 4, 8 12 (primary endpoint) and 52 weeks after the radiotherapy is completed. Survival data will be collected and assessed at the end of the study.

Brief Summary:

The purpose of this study is to investigate whether palliative radiotherapy delivered 1-2 fractions of 8 Gy is non-inferior to palliative radiotherapy delivered in 5 fractions of 5 Gy with respect to change in patient-reported symptom intensity in patients with pelvic soft tissue tumors originating from either gastrointestinal, urological or gynecological cancer. Patients will define a target symptom as their main complaint according to study-specific predefined categories (pain, bleeding, bowel dysfunction, lower urinary tract dysfunction or vaginal dysfunction).

Patients will be randomly assigned to radiotherapy intervention with either 1-2 fractions of 8 Gy or 5 fractions of 5 Gy. Patients entering the study must be considered as candidates for palliative radiotherapy, and have a target symptom intensity at baseline of minimum 4 points on the NRS, which ranges from 0-10 with 0 indicating no symptom intensity and 10 indicating the worst symptom intensity imaginable.

For patients randomized to 1-2 fractions of 8 Gy, the second fraction will be offered if predefined criteria for unsatisfactory symptomatic response are met, assessed at the 4-week follow-up (see Section 4.3.1).

Assessment of endpoints will be performed at 4, 8, 12 (primary endpoint), 26 and 52 weeks after the radiotherapy is completed. Assessments include patient-reported evaluation of symptom intensity (NRS), QoL, overall patient satisfaction and physician-reported toxicities. Subjects will be followed with respect to overall survival until death, or censoring, defined as either loss to follow-up, study withdrawal or end of study.

The radiotherapy planning and deliverance, and the study-specific follow-up visits (attendance or by telephone) will be performed at the patients` local radiotherapy unit. Additional follow-up visits related to the patients' cancer disease will be performed at their local cancer department, and will be according to the institutional practice, independent of study inclusion.

Patients will be included over a period of 2-4 years with a maximum follow-up at study closure (overall survival).

The results of this study may contribute to the establishment of standard treatment recommendations, both nationally and internationally. Both interventions are currently being used in clinical practice, which will facilitate the implementation of study results.

Number of Participants:

A maximum of 200 patients will be randomly assigned to study intervention to acheive a per protocol (PP) population of 146 patients

Data Monitoring/Other Committee:

The study is organized with a Data Monitoring Committee (DMC) and Steering Committee

1.2. Schedule of Activities (SoA)

Procedure	Up to 28 days		Intervention Period [1 or 5 weekdays]					Follow-up[a]						Notes
	**Screening**	**Preparation**	**D1**	**D2**	**D3**	**D4**	**D5**	**1 week +/- 3 days**	**4 weeks +/- 4 days**	**8 weeks +/- 4 days [b]**	**12 weeks +/- 7 days**	**26 weeks +/- 2 weeks**	**52 weeks +/- 4 weeks**	Follow-up, either phone call or visit
Informed consent	X													Section 10.1.2
Inclusion and exclusion criteria		X												Section 5
Demography and medical history	X													Section 8.1
ECOG performance status	X								X		X			Section 8.3.3
Pregnancy test (WOCBP)		X [c]												Section 8.3.5
Local laboratory tests (albumin, CRP, hemoglobin, platelets, LDH)	X													Section 8.4.1
Randomization		X[d]												Section 6.3
CT for radiotherapy planning	X								(X)[k]					Section 6.2
Cone beam CT (or kV/kV)									X[k]					Section 6.2

Procedure	Up to 28 days		Intervention Period [1 or 5 weekdays]					Follow-up[a]						Notes
	**Screening**	**Preparation**	**D1**	**D2**	**D3**	**D4**	**D5**	**1 week +/- 3 days**	**4 weeks +/- 4 days**	**8 weeks +/- 4 days [b]**	**12 weeks +/- 7 days**	**26 weeks +/- 2 weeks**	**52 weeks +/- 4 weeks**	Follow-up, either phone call or visit
Intervention/treatment, arm A			X						X[b,j]					Section 6.1, 4.3.1
Intervention/treatment, arm B			X	X	X	X	X							Section 6.1
NRS score target symptom	X								X	X	X	X		Section 8.2.1.1
NRS score other symptoms	X								X	X	X	X		Section 8.2.1.1
EORTC QLQ-C15 PAL		X							X	X	X	X		Section 8.2.1.2
Patient Global Impression of Change (PGIC)									X	X	X	X		Section 8.2.1.3
EQ-5D-5L		X							X	X	X	X		Section 8.2.3
CTCAE v.5.0		X							X		X		X	Section 8.3.2
GPS		X												Section 8.4.1
LabBM score		X												Section 8.4.1
LabPS score		X												Section 8.4.1

Procedure	Up to 28 days		Intervention Period [1 or 5 weekdays]					Follow-up[a]						Notes
	Screening	Preparation	D1	D2	D3	D4	D5	1 week +/- 3 days	4 weeks +/- 4 days	8 weeks +/- 4 days[b]	12 weeks +/- 7 days	26 weeks +/- 2 weeks	52 weeks +/- 4 weeks	Follow-up, either phone call or visit
AE review			←==================→					X[h]	X[h]	X[k,h]	X[i]		X[i]	Section 8.5
SAE review			←==================→					X[h]	X[h]	X[k,h]	X[i]		X[i]	Section 8.5
Supportive medications[e]	X								X		X			Section 6.8, 8.1
Radiotherapy record[f]	X										X		X	Section 6.6, 8.1
Administration[g]								X	X	X[k]				Section 8.2.3

Abbreviations: AE=Adverse Event, CT= Computed Tomography, CTCAE= Common Terminology Criteria Adverse Events, CRP= C-reactive protein, ECOG = Eastern Cooperative Oncology Group, EORTC QLQ-C15 PAL= European Organization for the Research and Treatment of Cancer Quality of Life Questionnaire-Core15 Palliative, GPS= Glasgow Prognostic Score, LDH=Lactate dehydrogenase, NRS=Numerical Rating Scale, SAE=Serious Adverse Event, WOCBP=Women of childbearing potential

[a] All follow-up is defined from the day of completed radiotherapy (arm A: D1, arm B: D5 or D6-10 if modifications, see Section 6.5)

[b] If a second fraction is offered (see Section 4.3.1), evaluation must be scheduled 4 weeks +/- 4 days after the second fraction is delivered,. For all other patients, evaluations must be scheduled 8 weeks +/- 4 days from baseline

[c] Within 72 hours before randomization

[d] Only if baseline NRS score of target symptom is available

[e] Including pain medication, tranexamic acid, anticoagulants, laxatives, antidiarrheal medication, antiemetics and steroids

[f] Radiotherapy treatment within the same planning target volume (PTV) (additional fraction after 4 weeks in arm A is not considered reirradiation)

[g] I.e. hospital submissions and coding record related to radiotherapy

[h] All AE ≥grade 3/SAE

[i] Only AE ≥grade 3/SAE with suspected causality with radiotherapy

[j] To be performed within 4 days after decision

[k] Only if second fraction is offered. See Section 4.3.1

2. Introduction

Palliative radiotherapy is cost-effective and time-efficient, and provides local symptom alleviation in patients with metastatic cancer. Radiotherapy research in general is though less prioritized (1), and patient-centered clinical endpoints, which are essential for the assessment of clinical benefit, are often lacking (1-5). Increased effort in palliative radiotherapy research, with emphasized focus on patient-centered and patient-reported outcomes, is required (1, 4, 6).

Clinical trials and clinical practice have demonstrated palliative radiotherapy to provide efficient symptom relief in patients with pelvic soft tissue tumors (2, 3, 7-16). Current state of the art is, however, based on mainly retrospective studies that are difficult to compare due to a variety of radiotherapy schedules used. Consequently, no recommended standard of care is established in Norway today, and the chosen radiotherapy schedule partially depends on cancer diagnosis and the institutional preference.

The prospective, multicenter, non-randomized PallRad study investigated the patient-reported symptomatic effect of a prolonged palliative radiotherapy schedule in patients with rectal and prostate cancer (8, 9). The study showed that radiotherapy provides efficient symptom relief with acceptable toxicities and good QoL, and displayed feasibility in addressing this particular topic in a multicenter setting in Norway.

Palliative patients have a limited life expectancy, favoring a short-course radiotherapy schedule. Further, in an oncologic landscape with expanding systemic treatment options, a short-course radiotherapy schedule, requiring a shorter break from systemic treatment, is applicable also in patients in need of both rapid local symptom relief and systemic disease control. Finally, a short-course radiotherapy schedule offers an optimal exploitation of health care resources. A short-course radiotherapy schedule has proven to be equally effective as more prolonged schedules in palliative patients with painful bone metastases (17-19) or thoracic symptoms from non-small cell lung cancer (20, 21). A randomized trial designed to provide evidence of the optimal radiotherapy schedule in patients with symptomatic pelvic soft tissue tumors, comparing a short-course radiotherapy schedule with a more prolonged schedule, is e highly warranted.

Optimal symptom control after palliative radiotherapy is measured in weeks to months, hence, patients with a very short life expectancy limited to a few weeks would likely not benefit from treatment (22). Clinicians tend to overestimate patient survival (22). Prognostic models based on clinical characteristics have been developed to aid survival prediction (22-25), but need validation in this specific patient population. In addition, predictive factors associated with radiotherapy resistance could provide useful information in patient management, however, their predictive value in palliative radiotherapy is unclear (26-30).

2.1. Study Rationale

The main objective is to establish whether palliative radiotherapy with 1-2 fractions of 8 Gy is non-inferior to 5 fractions of 5 Gy with respect to change in patient-reported target symptom intensity in patients with pelvic soft tissue tumors. If the main objective is achieved, and general QoL, toxicities and overall survival are comparable between the two study arms, a short-course schedule of 1-2 fractions may be recommended as standard of care, particularly beneficial for both patients with a short life expectancy and patients eligible for systemic treatment. A short-course radiotherapy schedule would also provide improved exploitation of the radiotherapy treatment capacity and reduce costs related to patient transportation and hospitalization.

2.1.1. Principal Research Question

The principal research question is whether radiotherapy with 1-2 fractions of 8 Gy (arm A) provide non-inferior patient-reported target symptom relief as compared to a more prolonged radiotherapy schedule with 5 fractions of 5 Gy (arm B) in patients with symptomatic pelvic soft tissue tumors, assessed after 12 weeks of follow-up. The target symptom is the patient-reported main complaint according to study-specific predefined categories (pain, bleeding, bowel dysfunction, lower urinary tract dysfunction or vaginal dysfunction).

2.1.2. Secondary Research Questions

The secondary research questions are whether the two study arms differ with respect to physician-reported bladder and bowel toxicities after 4, 12, and 52 weeks of follow-up and overall survival, respectively.

2.1.3. Exploratory Research Questions

The exploratory research questions are whether the two study arms differ with respect to the following:

- Change in QoL from baseline after 4, 8, 12 and 26 weeks of follow-up.
- Physician- reported overall toxicities after 4, 12 and 52 weeks of follow-up.
- Patient-reported target symptom relief after 4, 8and 26 weeks of follow-up.
- Patient-reported relief of symptoms related to the irradiated tumor, other than the target symptom, after 4, 8, 12 and 26 weeks of follow-up.
- Overall patient satisfaction after 4, 8,12 and 26 weeks of follow-up.

Clinical factors, hypoxia biomarkers and PD-L1- status may be explanatory variables of symptomatic effect

Prognostic models may be relevant for patient classification and treatment decision

Health economic aspects may differ between study arms.

2.1.4. Rationale for Timing of Endpoint Assessment

In the PallRad study, assessment of symptom relief was performed at the end of treatment, and after 6 weeks and 12 weeks follow-up (primary endpoint) (8, 9). Results showed an increase in proportion of responders from end of treatment until 6 weeks, but the proportion was relatively stable between 6 and 12 weeks. The point in time of the expected maximum radiotherapy response depends, among other things, on the target symptom to be treated, and it can extend up to 12 weeks after treatment completion. To ensure correct assessment of all patients, including late responders, the primary endpoint will be assessed at 12 weeks. An additional assessment of symptom relief will be performed also after 26 weeks.

The pragmatic design of this study allows systemic cancer treatments in close proximity to the radiotherapy, which, for some patients, may interfere with the response assessment at 12 weeks. However, due to the randomized allocation of patients, and the total sample size, we expect this to be evenly distributed across the study arms.

The dynamics of the symptomatic response will provide relevant information, and it is therefore important to include early assessments at 4 weeks and 8 weeks as exploratory endpoints. In the PallRad study, patients were treated with a dose of 3 Gy per fraction. Due to the higher doses per fractions planned in this study (8 Gy in arm A, 5 Gy in arm B), we anticipate a more rapid symptomatic response. Furthermore, patients in arm A with unsatisfactory response will be offered a second radiotherapy fraction. From a radiobiological perspective, the interval between fractions should be kept at a certain minimum. Assessment of the symptom relief at 4 weeks (secondary endpoint) therefore represents a reasonable compromise between a reliable response evaluation and radiobiological considerations.

2.2. Background

2.2.1. Background Disease

Soft tissue tumors in the pelvic region often originate from gastrointestinal, urological or gynecological primary cancers. They include primary tumors, local recurrences and metastases, and are often symptomatic. Regardless of origin, symptoms consist of pain, bleeding, or bowel/lower urinary tract/vaginal dysfunction. All these symptoms often severely affect QoL (31).

Studies and clinical practice have proven palliative radiotherapy to be an efficient treatment for patients with symptomatic pelvic soft tissue tumors (2, 3, 7-14). Due to the similar symptom burden, it is appropriate and practical to study palliative radiotherapy in these patients as one single group independent of disease origin.

In the sections below, cancers where symptomatic pelvic soft tissue tumors most frequently occur are briefly described. Common for all cancers is that patients unfit for curative treatment,

due to either advanced loco-regional/systemic disease, or medical contradictions such as high age, comorbidity and poor performance status, are candidates for palliative radiotherapy.

2.2.1.1. Rectal and Anal Cancer

Colorectal cancer is one of the most frequent cancers worldwide, with a high incidence in Norway (32) For rectal cancer, advances in diagnostics, staging, surgical techniques, radiotherapy and chemotherapy have resulted in a low local recurrence rate and an improved overall survival (33-36). The main curative treatment is surgery alone, or surgery in combination with radiotherapy and chemotherapy (34, 35).

Anal cancer is a relatively rare disease, but with an increasing incidence (37, 38). Most tumors (90%) are human papilloma virus (HPV) 16- positive, while HPV negative tumors carry a poor prognosis (39, 40). The main curative treatment is radical radiotherapy in combination with chemotherapy (41).

2.2.1.2. Urological Cancer

Prostate cancer is the most frequent cancer in Norwegian men, and accounts for approximately 30% of all male cancers (32). The prevalence is rising, but the mortality (32). The main curative treatment is surgery or radiotherapy in combination with anti-hormonal therapy (42)

Cancer of the bladder and urinary tract cancers occur most frequently in Norwegian men, accounts for approximately 7% of all male cancers and has a rising incidence (32). Over the last 50 years, survival has been steadily increasing (32). The main curative treatment for early stages is surgery, while locally advanced stages are preferably treated with surgery in combination with radiotherapy and chemotherapy (43).

2.2.1.3. Gynecological Cancer

Gynecological cancer originates from the ovaries, cervix, endometrium, vagina or vulva. The incidence of gynecological cancers in Norway has been fairly stable over the last decade, while the survival rate is slowly increasing (32). When diagnosed at an early stage, gynecological cancer generally carries a good prognosis (32). Cervical and endometrial cancers are often detected at early stages due to presenting symptoms like bleeding and pain, and due to the implemented cervical cancer screening program. For ovarian cancer, however, symptoms are often insidious, and due to lack of established screening methods, the disease often presents in more advanced stages (32).

In early stages, the primary treatment of all gynecological cancers is surgery (44-47), often combined with chemotherapy or radiotherapy in high-risk patients (45, 48-51). For locally advanced cervical, vaginal and vulvar cancers, radical radiotherapy in combination with

chemotherapy is the primary treatment (45, 52). In endometrial or ovarian cancer, surgery is also performed at locally advanced stages, often combined with chemotherapy (49, 53).

2.2.2. Background Treatment

2.2.2.1. Palliative Radiotherapy

The clinical benefit of palliative radiotherapy relies on its local cytotoxic effect allowing for arrest or regression of the irradiated tumor, and consequently a reduction of specific tumor-related symptoms. The goal is rapid and lasting symptom relief due to local tumor control, with limited acute and late side effects.

A higher total radiation dose increases the probability of local tumor control (54). Radiotherapy relies on the premise that the normal tissue has superior capacity to repair sub-lethal damage compared to the tumor, described as the therapeutic ratio. Radiotherapy delivered over a prolonged course with several fractions augments this ratio, and enables enhanced normal tissue cell repair in intervals between fractions. Consequently, a higher total radiation dose can be delivered to the tumor within the normal tissue tolerance (54, 55). The fractionation sensitivity varies between different tissues, and can be assessed by the linear-quadratic (LQ) model, and expressed by the alpha/beta (α/β) ratio (56).

Sufficient cell repair is particularly important in late-reacting normal tissue where exposure to radiation doses over certain threshold values can cause irreversible damage known as late side effects. However, in prolonged radiotherapy courses with several fractions, the interval between fractions simultaneously allows for tumor cell repopulation increasing the risk of local tumor control failure. Depending on the clinical situation, radiotherapy is therefore delivered with different fractionation schedules in order to yield the most optimal balance between tumor and normal tissue responses, tumor cell repopulation, and normal tissue repair (54, 55).

For palliative patients with a limited life expectancy, there is an accentuated importance of rapid symptom control and reduced overall treatment time. A short-course radiotherapy schedule with larger doses per fraction (i.e. hypofractionated radiotherapy) is therefore appealing. Although late side effects are less of a concern in palliative patients with a short life expectancy, advances in systemic cancer treatment and precision oncology challenge lifetime predictions. However, with modern radiotherapy techniques including Intensity-Modulated RadioTherapy (IMRT), Volumetric Modulated Arc Therapy (VMAT), and Image-Guided RadioTherapy (IGRT), high radiotherapy doses can be delivered to the tumor with simultaneous adequate sparing of surrounding normal tissue, reducing the risk of serious late side effects. At the most extreme, an adequate radiation dose can be delivered in only one fraction (unfractionated radiotherapy) with simultaneous control of relevant side effects.

A short-course radiotherapy schedule reduces time commitment and allocated resources at the radiotherapy unit, thereby improving the exploitation of treatment capacity. In addition, costs related to patient transportation and hospitalization are reduced.

In palliative patients with either painful bone metastases or thoracic symptoms from non-small cell lung cancer, a short-course radiotherapy schedule of 1 fractions or 2 fraction, respectively, has proven to be equally effective and tolerable as prolonged schedules (17, 57-59). Also, for rectal cancer, a short-course radiotherapy schedule of five fractions has proven tolerable and with efficacy in elderly patients and patients with metastatic disease (60, 61). However, for patients with symptomatic pelvic soft tissue tumors, the optimal radiotherapy schedule is yet to be established (2, 3, 7, 13, 14).

Prognostic models for patient classification

Patients who receive palliative radiotherapy represent a heterogeneous group with great differences in overall survival. Optimal symptom control after palliative radiotherapy is measured in weeks to months, and patients with a very short life expectancy limited to a few weeks, would likely not benefit from treatment (22). Clinicians tend to overestimate patient survival (22). Prognostic models based on clinical characteristics have been developed to aid survival prediction (22-25, 62), but further validation in the current study population is necessary. The prognostic scores GPS, LabBM, and LabPS are based on blood samples and information on the patients' general condition, and will be assessed

GPS	C-Reactive Protein (CRP), albumin
LabBM	CRP, albumin, lactate dehydrogenase (LDH), hemoglobin, platelets
LabPS	CRP, albumin, LDH, hemoglobin, platelets and Eastern Cooperative Oncology Group (ECOG) Performance Status

Predictive biomarkers;

The integration of imaging- and biopsy-derived biomarkers in a clinical trial investigating palliative radiotherapy, where symptomatic effect is more important than the absolute tumor shrinkage, is a novel, translational approach that will provide additional knowledge of potential value for treatment decisions in individual patients

Tumor hypoxia:

Tumor hypoxia, a state with depleted oxygen tension in the tumor, is as an aggressive feature of solid tumors that is associated with all of the cancer hallmarks (29), and it promotes tumor growth, invasion and metastasis (63, 64). Moreover, tumor hypoxia below certain levels is associated with significant resistance to radiotherapy (29, 65). Clinically feasible tools for

hypoxia assessment based on MRI (27, 66) and expression of hypoxia-activated genes in tumor biopsies have been developed (28, 67), which allows for patient classification based on hypoxia status. The significance of tumor hypoxia in the response to palliative radiotherapy is currently unknown.

PD-L1 status:

Programmed death-ligand 1 (PD-L1), a transmembrane protein that is often upregulated in cancer cells, and plays a key role in tumor immunosuppression (68). PD-L1 expression is assessed by immunohistochemistry, and indicated as either a total proportion score (TPS) or combined positive score (CPS) (69). Radiotherapy modulates the tumor immunity (70), and PD-L1 status has been shown to be a predictor of the radiotherapy response (30), which might also be of relevance in palliative radiotherapy.

Health economic aspects

A short-course radiotherapy schedule is advantageous in a health economic perspective, as costs related to transportation to and attendance at the radiotherapy unit, are reduced. In addition, a short-course schedule may reduce costs related to elective hospital submissions of patients that are uneligible for repeated daily transportation.

2.2.2.2. Rectal and Anal Cancer

A systematic review of palliative pelvic radiotherapy for symptomatic, incurable rectal cancer included mostly retrospective studies using a variety of radiotherapy schedules ranging from 5 to 70 Gy in total radiation doses (3). The studies reported good symptomatic response, but lacked patient-reported and QoL measures. Toxicity results were not evaluable.

In the PallRad study, including patients with rectal cancer, Cameron et al. reported an overall target symptom response of 85% at both the 6-week and the 12-week follow-up visits (8). The QoL remained stable throughout the study period. Patients were treated with a prolonged course of 10-13 fractions, yielding a total dose of 30-39 Gy. The median survival time in the study was 9 months, underlining the limited life expectancy in palliative patients and the importance of reduced overall treatment time.

Another study, including patients with non-resectable or metastatic rectal cancer, evaluated the effect of a short course radiotherapy schedule of 5 Gy in 5 fractions followed by oxaliplatin-based chemotherapy (71). The main outcome was the avoidance of surgery (tumor resection or stoma), and was achieved in 80% of patients. A proportion of 35% of patients had a complete resolution of pelvic symptoms, and 35% had a clinically significant improvement.

In a study on inoperable/elderly patients with rectal cancer, patients were treated with external beam radiotherapy of 39 Gy in 3 Gy fractions in combination with high-dose-rate rectal brachytherapy (72). The study reported good tumor response but a high rate of toxicity.

To our knowledge, there are no studies on palliative radiotherapy of anal cancer.

2.2.2.3. Urological Cancer

A systematic review of palliative pelvic radiotherapy for symptomatic, incurable prostate cancer included only retrospective studies employing a large variation of radiotherapy dose and schedules (2). The studies reported good symptomatic response, but lacked reports on patient-reported or QoL measures, and toxicity results were not evaluable.

In the PallRad study, including patients with prostate cancer, Cameron et al. reported an overall target symptom response of 80% and 70 % at the 6-week and 12-week follow-up visits, respectively (9). The global QoL transiently improved during the study. Patients were treated with a prolonged course of 10-13 fractions, yielding a total dose of 30-39 Gy. The median survival time in the study was 20 months.

In the prospective, randomized BA09 trial from 2000, two schedules of palliative radiotherapy (35 Gy in 10 fractions and 21 Gy in 3 fractions) were compared in patients with symptomatic bladder cancer. The study found the two schedules to be equally effective with respect to symptomatic improvement, and toxicities were comparable (13). Moreover, in a systematic review on bladder cancer, the authors concluded that palliative radiotherapy provides excellent and rapid symptom relief, particularly in patients with symptomatic hematuria, and that a short course-schedule is preferable (14).

2.2.2.4. Gynecological Cancer

In an observational, retrospective study from 1986, Halle et al. studied the effect of a single fraction of 10 Gy to the pelvis in advanced cancers of the cervix or endometrium (73). The fraction was repeated once or twice at monthly intervals when necessary. The study reported acceptable symptomatic response, but serious side effects were observed in patients surviving more than approximately one year. The authors therefore concluded that 10 Gy fractions should be limited to patients with a life expectancy of less than 12 months.

A similar retrospective Norwegian study, also employing a schedule with one single 10 Gy fraction, was conducted in 2001, and included patients with a life expectancy of less than 12 months (74). However, 28 % of the patients survived beyond 12 months, demonstrating the challenge with lifetime predictions. The study reported a good response on bleeding and vaginal discharge, but serious bowel complications occurred from 9 months onward after radiotherapy.

In a more recent, retrospective study from 2011, another regimen with a single fraction of 8 Gy, repeated at day 7 and 21 if feasible and necessary, was used (75). The study reported effective and rapid symptomatic responses with acceptable toxicity.

2.2.2.5. Pelvic Cancer

In the prospective, randomized RTOG 8502 trial from 1994, 290 patients with all types of advanced symptomatic pelvic cancer were included (76). To maintain similar tumor response as reported in previous studies on gynecological cancer using short-course schedules with high doses per fraction while trying to reduce serious side effects, an alternative fractionation schedule was employed where patients received 3.7 Gy twice daily for a two-day course to a total of 14.8 Gy. If feasible and necessary, the treatment was repeated at four-week intervals for a maximum of three cycles. Similar tumor response rates as the previous studies were reported, and fewer late complications were observed.

In a prospective study from 2019 including 25 patients with symptomatic pelvic tumors, a short-course radiotherapy schedule of 4.5 Gy twice daily in two days, was tested (10). An overall palliative response of 96% was found, with a median duration of response of 6 months. Two thirds of patients reported either stable or improved QoL. The study resulted in an ongoing phase III randomized clinical trial comparing the symptomatic effect of the short-course schedule with a prolonged two-week schedule (ClinicalTrials.gov registry number: NCT03804333).

2.3. Benefit/Risk Assessment

This is a non-inferiority study aiming to show that the shorter course of radiotherapy is non-inferior to a more prolonged course of radiotherapy in symptomatic pelvic soft tissue tumors. Both radiotherapy courses are currently used in routine clinical practice for this indication. Based on clinical experience, both radiotherapy courses provide good symptom relief and are well tolerated.

The radiotherapy will be delivered according to the institutional practice of each participating site without study-specific adjustments. Study-specific procedures applied on patients include questionnaires (symptomatic response, QoL, general satisfaction and toxicities) and laboratory test results. The required laboratory tests are included in the routine patient follow-up for assessment of their general condition, and will therefore not be considered as study-specific. As baseline patient-reported symptom intensity is decisive for indication of radiotherapy, baseline symptom assessmen is considered part of clinical routine..

The treatment efficacy and toxicities will be closely monitored according to the SoA (Section 1.2). The patients will have daily contact with the radiation therapists at the respective radiotherapy units during the study intervention for reporting of toxicities or other complaints. If the patient`s general condition deteriorates, the radiation therapists will notify either an oncologic nurse or the clinical oncologist who will assess the patient and initiate relevant interventions. To ensure collection of all acute AEs (≥ grade 3) and SAEs during intervention, a study specific follow-up with either a clinical oncologist or a study nurse is scheduled (Section 1.2).

The 4-week follow-up will assure collection of acute AE (≥ grade 3) and SAE after the intervention. From the 4-week to the 52-week follow-up, only AE (≥ grade 3) and SAE with probable/definite causality with radiotherapy will be reported

Safety will be evaluated by a DMC (Appendix 10.1.4)

Medical images and biopsies that are acquired for diagnosis or treatment will be analyzed for exploratory endpoints in available patients. There will not be performed study-specific patient procedures for these endpoints.

3. Objectives, Endpoints and Assessments

Primary objective

Assess whether 1-2 fractions of 8 Gy is non-inferior to 5 fractions of 5 Gy with respect to target symptom relief.

Endpoint: Change in the average (last 24 hours) patient-reported target symptom intensity from baseline (BL) assessed after 12 weeks of follow -up

Assessment: Numerical Rating Scale (NRS) score (0-10)

Secondary objective

To assess the efficacy of 1-2 fractions of 8 Gy compared to 5 fractions of 5 Gy with respect to:

- Physician-reported toxicities

  Endpoint: Bladder and bowel toxicity after 4, 12 and 52 weeks of follow-up

  Assessment: Common Terminology Criteria for Adverse Events (CTCAE) v 5.0

- Survival

  Endpoint: Overall survival (OS) assessed at the end of study

Exploratory objectives

1. *To assess the efficacy of 1-2 fractions of 8 Gy compared to 5 fractions of 5 Gy with respect to:*

- Patient-reported QoL

  Endpoint: Change in QoL from BL after 4, 8, 12 and 26 weeks of follow-up

  Assessment: European Organization for the Research and Treatment of Cancer Quality of Life Questionnaire-Core15 Palliative (EORTC QLQ-C15 PAL)

- Physician-reported toxicities

  Endpoint: All relevant toxicities after 4, 12 and 52 weeks of follow-up

  Assessment: CTCAE v 5.0

- Patient reported symptomatic effect

  Endpoints: Change in target symptom intensity from baseline after 4 weeks and 26 weeks of follow-up, and changes in intensity of symptoms related to the irradiated tumor other than target symptom (within the five predefined categories) from baseline after 4, 8, 12 and 26 weeks of follow-up

  Assessment: NRS score 0-10

- Patient satisfaction

  Endpoint: Assessment of patient satisfaction after 4, 8, 12 and 26 weeks of follow-up

  Assessment: Patient Global Impression of Change (PGIC)

2. To explore prognostic models for patient classification

Endpoint: Correlation between prognostic scores assessed at baseline and overall survival

Assessment: Glasgow Prognostic Score (GPS), LabPS- score and LabBM- score

3. To explore clinical factors as explanatory response variables

Endpoint: Correlation between clinical factors and symptomatic response

Assessment: Baseline registration of clinical factors from medical records

4. Assess hypoxia biomarkers on medical images and biopsies that are previously acquired for diagnosis or treatment, and explore their potential as explanatory response variables in individual patients.

Endpoint: Correlation between hypoxia biomarkers and symptomatic response

Assessment: Hypoxic fraction assessed by Consumption and Supply-based Hypoxia (CSH)-imaging on MRI (Magnetic Resonance Imaging) and hypoxia-activated protein and gene expression assessed by molecular analyses on tumor biopsies

5. Assess PD-L1 (Programmed Death-Ligand1)-status in biopsies previously acquired for diagnosis or treatment, and explore potential as explanatory response variable.

Endpoint: Correlation between PD-L1-status and symptomatic response

Assessment: PD-L1 expression level (all cells) assessed by immunohistochemistry

6. Compare the health-economic aspects in the two study arms regarding

- *Patient-reported QoL*

  Endpoint: Change in QoL compared to baseline assessed after 4, 8,12 and 26 weeks

  Assessment: EQ-5D-5L (5 Level-EuroQol-5 Dimensions)

- *Health service costs*
  Endpoint: Difference in costs related to attendance at the radiotherapy unit, transportation and hospital submissions
  Assessment: Registration of costs from medical records

4. Study Design

4.1. Overall Design

This is a prospective, randomized, open-label, national, parallel-arm non-inferiority phase III study designed to establish nin-inferiority of radiotherapy with 1-2 fractions of 8 Gy to 5 fractions of 5 Gy (active comparator) with respect to patient-reported symptomatic relief. The study population includes patients with gastrointestinal, urological, or gynecological cancer considered as candidates for palliative radiotherapy due to symptomatic pelvic soft tissue tumors. Patients must have an expected survival beyond 12 weeks. Patients will be included over 2-4 years. Eligible patients identified in the screening period will be randomized to either arm A or arm B with an allocation ratio of 1:1 after collection of a signed informed consent. Study-specific baseline evaluation will be performed between date of signed inform consent and intervention start.

Patients in arm A will initially be treated with a single fraction of 8 Gy. In case of unsatisfactory symptom relief after 4 weeks of follow-up, a second radiotherapy fraction may be offered if at least one of the following two predefined criteria are met: 1) ≤2 point improvement on the NRS-scale and 2) initial symptomatic effect, but rapid deterioration (Section 4.3.1). Primary and secondary endpoints will be assessed after 4, 8, 12, 26 and 52 weeks of follow-up (last visit, toxicities only) after completed radiotherapy. Subjects will be followed with respect to overall survival until death or censoring (loss to follow-up, study withdrawal, or end of study).

The study timeline is presented in Figure 1.

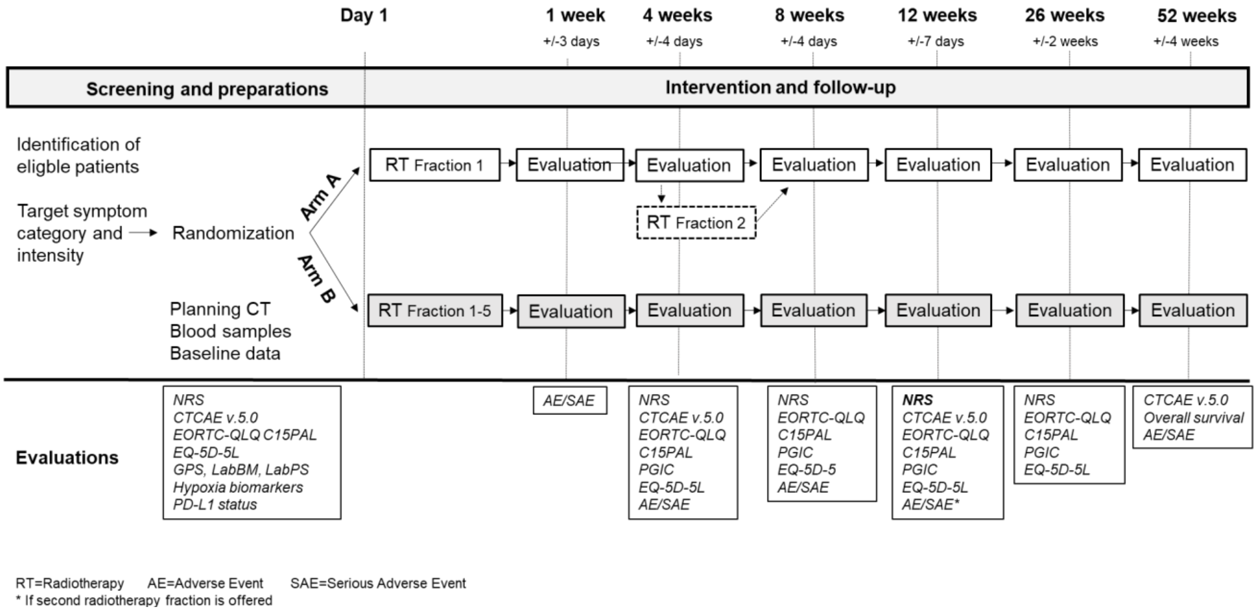

Figure 1: Study timeline

4.2. Scientific Rationale for Study Design

The principal research question is whether a short-course radiotherapy schedule of 1-2 fractions provide non-inferior patient-reported symptom relief compared to a schedule of 5 fractions, assessed 12 weeks after completed radiotherapy. If non-inferiority is established, and general QoL, toxicities and overall survival are comparable between the two study arms, a short-course schedule will be preferred, as it will be less burdensome for patients and provide improved exploitation of health care resources. A superiority study would therefore not be necessary in order to implement treatment results. Although superiority studies that fail to reject the null hypothesis are frequently interpreted as showing no differences between groups, they should be interpreted as indeterminate (77).

If non-inferiority can be proved only 4 weeks after completed radiotherapy, a single fraction schedule will be recommend for patients with a very short life expectancy for whom palliative radiotherapy is considered reasonable.

4.2.1. Participant Input into Design

A user representative is included in the study group, and has contributed to the preparation of the informed consent form, study questionnaires and study protocol. The user representative will be invited to the overall study group meetings.

4.3. Justification for Radiotherapy Dose

4.3.1. Second Radiotherapy Fraction in Arm A

For patients in arm A receiving an initial single fraction of radiotherapy, a second fraction *may* be offered at the 4-week follow up visit (attendance or telephone) if the clinical oncologist and the patient find it appropriate, and if at least one of the following occurs:

- Unsatisfactory symptom relief, defined as ≤2 point improvement on the NRS scale compared to baseline
- Initial symptomatic effect, but rapid deterioration of symptoms

If systemic treatment is resumed after the first fraction (Section 5.1 and 6.8), and the patient is scheduled for a second fraction after the 4- week follow-up visit, systemic treatment must be paused from the second fraction is determined until one week after completed radiotherapy.

Patients in both study arms may not be included in other interventional clinical trials until the 4 week assessment is completed (Section 6.8).

4.4. End of Study Definition

The end of study is defined as the last patient last visit (LPLV), i.e. the data when the last patient complete the study. A patient is considered to have completed the study if he/she has completed all phases of the study including the last visit at 52 weeks.

The study may be terminated by the investigators, trial steering committee and/or sponsor for clinical or administrative reasons.

The end of study will be reported to the Regulatory Authority and institutional review boards (IRB)/independent ethics committee (IEC) within 90 days, or 15 days if the study is terminated prematurely. The investigators will inform included patients of the premature end of study and ensure that appropriate follow-up is arranged for all patients involved

5. Study Population

Patients will be recruited from their local oncology department or palliative unit, and must be considered as candidates for palliative radiotherapy according to the two study arms by their treating physician. To minimize the total burden on the patient, the recruitment procedure is customized to standard clinical practice in order to limit additional attendances at the hospital.

If all inclusion and exclusion criteria are met, patients will be informed about the study by their treating physician or nurses, and asked to participate. A written informed consent form will be handed out. If the patient is interested in participation, the patient will be contacted by a study nurse after sufficient time to consider participation. This contact may be in connection with a planned attendance at the hospital, or by telephone. Sufficient time to consider participation include time to read, understand and reflect upon the written informed consent form, as well as to have time to consult nest of kin or primary physician regarding potential participation. If the patient is contacted by telephone, information about the specific telephone number and the date and time of the telephone call will have been provided in advance. There will be allocated enough time in order to answer all potential questions the patient might have. If the patient wants to participate, the signed informed consent will be collected in connection with a planned attendance at the hospital. This attendance will be scheduled by the study nurse, and may often be in connection with the standard radiotherapy planning procedures.

To ensure inclusion of all eligible patients, participants may also be identified by study personnel allocated to the radiotherapy unit, after referral to radiotherapy by the treating physician. Study personnel at the radiotherapy unit will be indicated on the study delegation log, but will not have a dedicated treatment responsibility towards the patient. If possible participants are identified, study eligibility will be discussed with the treating physician and PI. If considered eligible for inclusion, patients will receive the written informed consent form by post or Helse Norge in advance, and will subsequently be informed about the study at the planned attendance scheduled for radiotherapy planning procedures. If a possible participant is identified the same day as this planned attendance, study personnel or the treating physician will first inform about the study in connection with the attendance, and the written informed consent form will subsequently be handed out. If the patient is interested in participation, further procedures will be in accordance with the description in the section above.

5.1. Inclusion Criteria

- Histologically verified primary cancer originated from gastrointestinal, urological or gynecological organs (histological verification can be performed on other lesions than the symptomatic pelvic tumor)

- Patients unsuitable for curative treatment due to either advanced disease or medical contradictions (i.e. comorbidity, old age or poor general condition)
- Primary, residual, recurrent or metastatic pelvic tumor from the abovementioned cancers not amenable for curative treatment
- Tumor-related symptoms within the following five predefined categories: pain, bleeding, bowel dysfunction, lower urinary tract dysfunction and vaginal dysfunction
- Considered as a candidate for palliative radiotherapy according to both study arms
- Patient reported average severity of symptoms ≥4 on a NRS scale of 0-10
- ≥18 years of age
- Speaks and understands Norwegian or English
- Ability to understand and willing to sign a written informed consent form
- ECOG performance status of 0-3
- Expected survival > 12 weeks
- Able to pause systemic cancer treatment for one week prior to, during, and one week after radiotherapy
- Women of childbearing potential (WOCBP) should have a negative highly sensitive serum pregnancy test within 72 hours prior to randomization. WOCBP must agree to the use of highly effective birth control methods or abstain from heterosexual sexual activity from randomization until completed study intervention

5.2. Exclusion Criteria

- Neuroendocrine histology of any kind
- Sarcoma or sarcomatous components in the histology
- Tumors that originate from bony metastases without a soft tissue component, or tumors if which the soft tissue component constitutes less than 50% of the total tumor volume, or if the soft tissue component is unlikely to contribute to the target symptom

- Unable to complete study questionnaires
- Ongoing treatment with an investigational drug at inclusion
- Planned inclusion in another interventional clinical study within 4 weeks after radiotherapy
- Pregnancy (due to risk of teratogenic and abortifacient effects of radiotherapy)

5.3. Lifestyle Considerations

Current smoking will be recorded in the electronic case report form (eCRF) as it can affect the radiotherapy response. However, no restrictions regarding smoking will be applied. No other lifestyle considerations are applicable.

5.4. Screen Failures

Screen failures are defined as patients who consent to participate in the clinical study but are not subsequently randomized to either of the study arms. A minimal set of screen failure information is required to ensure transparent reporting of screen failure participants to meet the Consolidated Standards of Reporting Trials (CONSORT) publishing requirements and to respond to queries from regulatory authorities. Minimal information includes demography, screen failure details, eligibility criteria, and any SAE.

Patients who do not meet the inclusion criterion of ≥4 points on a NRS- scale of 0-10 may be rescreened at a later point if symptoms deteriorates.

5.5. Criteria for Temporarily Delaying

If the intervention in arm B must be paused due to toxicities or other concerns, a maximum radiotherapy period of 10 weekdays is allowed. The decision of temporary treatment delay relies upon the local clinical oncologist.

6. Study Intervention(s) and Concomitant Therapy

Study intervention is defined as any investigational intervention(s), marketed product(s), placebo, or medical device(s) intended to be administered to a study participant according to the study protocol.

The study intervention in this study is radiotherapy delivered in two different schedules

6.1. Study Intervention

	Arm A		Arm B
**Intervention Name**	Radiotherapy		Radiotherapy
**Dose/fraction**	8 Gy		5 Gy
**Total number of fractions**	1	2	5
**Fractions per day**	1	1	1
**Total radiation dose in EQD2**[a]<br>**Tumor** (α/β of 10)<br>**Normal tissue (**α/β of 3)	 12 Gy<br>17.6 Gy	 24 Gy<br>35,2 Gy	 31.25 Gy<br>40 Gy

[a] Equivalent dose in 2 Gy fractions

6.2. Radiotherapy planning

The radiotherapy planning and delivery will be performed according to institutional practice at each participating site without study specific adjustments. However, contouring of nearby surrounding normal tissue/organs will be required as defined in the table below.

If a second fraction is offered to patients in arm A (section 4.3.1), a cone beam Computed Tomography (CBCT)is recommended for control of positioning. Alternatively, kV/kV may be applied if decided sufficient. The date of the CBCT (or kV/kV) should be recorded in the eCRF. If the local clinical oncologist considers replanning necessary, the reason for this should be recorded in the eCRF.

The following dose/volume parameters should be recorded in the eCRF.

	Volume (cm^3)	Dose (Gy) (nominal)	Definition
**Target**	GTV		Gross tumor volume
	CTV	CTV D98 (Gy)	Clinical target volume
	PTV	PTV D95 (Gy)<br>PTV D_{min} (Gy) (point dose)<br>PTV D_{max} (Gy) (point dose)	Planning target volume
**OAR**	Bladder	Average/mean dose (Gy)<br>$D_{max\ (0.1\ cm^3)}$ (Gy)	Whole organ
	Rectum	Average/mean dose (Gy)<br>$D_{max\ (0.1\ cm^3)}$ (Gy)	From top of anal canal to the rectosigmoid transisition
	Anal canal	Average/mean dose (Gy)<br>$D_{max\ (0.1\ cm^3)}$ (Gy)	3 cm from anus in axial plane
	Bowel bag	Average/mean dose (Gy)<br>$D_{max\ (0.1\ cm^3)}$ (Gy)	Intraperitoneal space from 2 cm above the upper limit of PTV to 2 cm below lower limit of PTV in axial plane

If target volume has a skeletal component (below 50 % according to exclusion criteria), this will be recorded in the eCRF.

6.3. Measures to Minimize Bias: Randomization and Blinding

During the screening period, patients will be assigned a unique identity number in the screening log in ascending numerical order at each study site. At randomization, each patient will be assigned a unique treatment number (randomization number) in the eCRF.

Treatment number encodes the patient’s allocation to one of the two study arms according to the randomization schedule.

This is an open-label study. Potential biases will be reduced by central randomization. Patients will be randomly assigned to one of the radiotherapy courses by an allocation ratio 1:1.

Randomization will be conducted using stratified block randomization by target symptom.

6.4. Study Intervention Compliance

Patients will only receive study intervention at the radiotherapy unit by designated staff (radiation therapists) and under medical supervision. The date and time of each radiotherapy fraction will be recorded in the source documents using radiotherapy software (OIS=Oncology Imaging System) at each unit. The correct study intervention (radiation dose per fraction and number of fractions) and the study participant identification, will be confirmed at the time of the first radiotherapy fraction by a member of the study site staff or a designated radiation therapist.

6.5. Radiotherapy Treatment Course Modification

The radiotherapy course of patients in arm B can be modified during the intervention if considered necessary by the local clinical oncologist. Modifications include fractionation changes or intervention pauses, and may be initiated as a result of extensive toxicity, deterioration in the general condition of the patient or poor patient compliance. In case of fractionation changes, the total nominal radiation dose received and number of radiotherapy fractions will be recorded in the eCRF. The patient may continue participation in the study if appropriate. If the radiotherapy is paused, a maximum radiotherapy duration of 10 weekdays is allowed, otherwise the intervention must be recorded as incomplete in the eCRF. A patient can resume intervention if considered appropriate by the local clinical oncologist.

If radiotherapy schedule modifications are deemed necessary prior to radiotherapy start in either study arm, the patient must be discontinued from the study.

6.6. Continued Access to Study Intervention after the End of the Study

After the 12-week follow-up visit, patients will resume to ordinary follow-up visits at their local cancer department. Patients may receive additional radiotherapy treatment within the same PTV after completion of study-specific follow-up visit at 4 weeks, but the following should be recorded in the eCRF:

- Radiation treatment within the same PTV until the 52 week follow-up visit.

6.7. Treatment of Overdose

Not applicable

6.8. Concomitant Therapy

Supportive medications will be recorded in the eCRF (Section 8.1).

Systemic cancer treatment must be paused for one week prior to, during, and one week after the radiotherapy treatment.

Patients cannot be included in other interventional clinical trials until the 4-week follow-up assessment is completed.

7. Discontinuation of Study Intervention and Participant Discontinuation/Withdrawal

7.1. Discontinuation of Study Intervention

It may be necessary for patients in arm B to permanently discontinue study intervention (i.e. radiotherapy), (Section 6.5). If study intervention is permanently discontinued, the patient may remain in the study to be evaluated for the planned assessments if the patient has not withdrawn the written consent. See the SoA (Section 1.2) for data to be collected at the time of discontinuation of study intervention and follow-up and further evaluations.

7.1.1. Temporary Discontinuation

See Section 6.5

7.1.2. Rechallenge

See Section 6.5.

7.2. Participant Discontinuation/Withdrawal from the Study

- A patient may withdraw from the study at any time at his/her own request or may be withdrawn at any time at the discretion of the investigator for safety, behavioral, or compliance reasons. This is expected to be uncommon.
- At the time of discontinuing from the study, if possible, an early discontinuation visit should be conducted, as shown in the SoA. See SoA (Section 1.2) for data to be collected at the time of study discontinuation.
- The patient will be permanently discontinued from both the study intervention and the study at that time.
- If the patient withdraws the consent for disclosure of future information, the sponsor may retain and continue to use any data collected before such a withdrawal of consent.
- If a patient withdraws from the study, he/she may request destruction of any samples taken and not tested, and the investigator must document this in the site study records.

7.3. Lost to Follow-up

A patient will be considered lost to follow-up if he/she repeatedly fails to return for scheduled visits and is unable to be contacted by the study site.

The following actions must be taken if a patient fails to return to the clinic for a required study visit:

- The site must attempt to contact the patient and reschedule the missed visit as soon as possible and counsel the patient on the importance of maintaining the assigned visit schedule and ascertain whether or not the patient wishes to and/or should continue to participate in the study.
- Before a patient is deemed as being lost to follow-up, the investigator or designee must make every effort to regain contact with the patient (when possible, telephone calls and, if necessary, a certified letter to the patient's last known mailing address or local equivalent methods). These contact attempts should be documented in the patient's medical record.
- Should the patient continue to be unreachable, he/she will be considered to have withdrawn from the study.

Discontinuation of specific sites or of the study as a whole is handled as part of Appendix 1.

8. Study Assessments and Procedures

- Study procedures and their timing are summarized in the SoA (Section 1.2). Protocol waivers or exemptions are not allowed.
- Immediate safety concerns should be discussed with the sponsor immediately upon occurrence or awareness to determine if the patient should continue or discontinue study intervention.
- Adherence to the study design requirements, including those specified in the SoA (Section 1.2), is essential and required for the study conduct.
- All screening evaluations must be completed and reviewed to confirm that potential study participants meet all eligibility criteria. The investigator will maintain a screening log to record details of all patients screened and to confirm eligibility or record reasons for screening failure, as applicable.
- Procedures conducted as part of the patient's routine clinical management (e.g., blood count) and obtained before signing of the informed consent form may be utilized for screening or baseline purposes provided the procedures met the protocol-specified criteria and were performed within the time frame defined in the SoA (Section 1.2).

8.1. Baseline Demography and Current Medical History

The following medical history will be recorded in the eCRF;

- Birthyear
- Sex
- Smoking status
- ECOG performance status
- Preferred language (Norwegian or English)
- Cancer diagnosis and disease status (primary origin only, locoregional disease below common iliac artery, locoregional disease below aortic bifurcation, metastatic)
- Histology and date of diagnostic/initial tumor biopsy
- Date of other (most and second most recent) tumor biopsies if performed
- Target symptom (indication for radiotherapy) and patient-reported symptom intensity
- Relevant comorbidity
- Concomitant supporting medications
    - Pain medication, opioid and non-opioid, invasive
    - Tranexamic acid
    - Anticoagulants
    - Laxatives
    - Antidiarrheal medication
    - Steroids
    - Antiemetics
- Systemic cancer treatment ongoing or in close proximity (withinlast 6 weeks)

- Relevant medical occurrences that begin before the start of study intervention but after obtaining informed consent. Relevant medical occurrences include those of possible impact on either of study endpoints (e.g. symptomatic effect, toxicities, QoL)
- Prior radiotherapy with overlapping radiotherapy field (re-irradiation)
- Baseline blood tests
- Date of prior MRI of tumor areas if performed
- If off study, date and cause
- If dead, cause of death
- If dead to other than cancer progression, autopsy performed

8.2. Efficacy Assessments

Planned time points for all efficacy assessments are provided in the SoA (Section 1.2).

8.2.1. Patient Reported Outcome Measures

8.2.1.1. Symptom Intensity

Patients will have a target symptom as indication for radiotherapy. Target symptom is the patients main complaint, and will be organized into five predefined categories (pain, bleeding, bowel dysfunction, lower urinary tract dysfunction, vaginal dysfunction). If present, two additional symptoms may be assessed.

Symptom intensity will be reported at baseline as part of clinical routine for decision regarding radiotherapy indication. Patients may require supportive medication adjustments to relieve symptoms pending on radiotherapy. Symptom intensity must be assessed *before* such adjustmenst, and adjustments shall be performed without any delay.

Symptom intensity will further be assessed after 4, 8, 12 and 26 weeks of follow-up.

Supportive medication corresponding with NRS at all timepoints will be recorded in the eCRF.

Symptom intensity will be measured on a NRS ranging from 0-10, where 0 (no symptom intensity) to 10 (worst symptom intensity imaginable). The following will be measured:

- Average symptom intensity the last 24 hours (primary endpoint)
- Maximum symptom intensity the last 24 hours
- Minimum symptom intensity the last 24 hours

Patients referred from hospitals without radiotherapy might have required adjustments in supportive medications prior to evaluation at the radiotherapy unit. Hence, these adjustments were performed before the patient was considered eligible for study inclusion, but may have reduced target symptom intensity below the limit for inclusion (NRS ≥4). In these cases, the target symptom intensity prior to adjustments in supportive medications may be registered as baseline NRS as long as registered supportive medications corresponds

8.2.1.2. EORTC QLQ-C15 PAL

The European Organization for Research and Treatment of Cancer Quality of Life Questionnaire- Core15-PAL.

Assessed at baseline and after 4, 8, 12 and 26 weeks of follow-up

8.2.1.3. PGIC 7-point scale

Patient global impression of change assessed on a 7-point scale.

Assessed after 4, 8, 12 and 26 weeks of follow-up.

8.2.2. Overall Survival

Will be assessed at the end of the study defined as LPLV.

8.2.3. Health economics

The 5 Level-EuroQol-5 Dimension (EQ-5D-5L) assessed at baseline and after 4, 8, 12 and 26 weeks of follow-up.

Costs related to attendance at the radiotherapy unit, transportation and hospitalization (including coding)

8.3. Safety Assessments

Planned time points for all safety assessments are described in the SoA (Section 1.2).

8.3.1. Follow-up after 1 week

A follow-up after one week for assessment of AEs (≥grade 3) and SAEs will be performed. Patients will have daily contact with radiation therapists at the radiotherapy unit during the study intervention for reporting of toxicities and other complaints.

8.3.2. Physician-assessed toxicity CTCAE

Common Terminology Criteria for Adverse Events version 5.0.

Assessed baseline and after 4 weeks, 12 weeks and 52 weeks of follow-up.

8.3.3. ECOG Performance Status

The Eastern Cooperative Oncology Group Performance Status (PS).

Assessed at baseline and after 4 and 12 weeks of follow-up

8.3.4. Clinical Safety Laboratory Assessments

Study-specific clinical safety laboratory assessments will not be performed. Relevant tests will be decided and acted on by the local clinical oncologist and will depend on the medical condition of individual patients. Such tests will not be recorded in the eCRF unless relevant for the reporting of AEs/SAEs.

Study-specific laboratory assessment included in prognostic scores (CRP, LDH, albumin, hemoglobin and platelets) will be performed (Section 8.4.1).

8.3.5. Pregnancy Testing

- To be performed before randomization of WOCBP. A woman is considered being of childbearing potential, i.e. fertile, following menarche and until becoming postmenopausal unless permanently sterile (due to hysterectomy, bilateral salpingectomy and bilateral oophorectomy), or considered infertile due to earlier cancer treatment.
- Due to the short intervention time, pregnancy testing will not be repeated during the study period.
- WOCBP must consent to the use of highly effective methods for birth control, be surgically sterile (Appendix 10.4) or abstain from heterosexual activity from randomization until completed study intervention (Section 5.1)

8.4. Other Assessments

8.4.1. Baseline Prognostic Scores

As an exploratory objective, the prognostic impact of GPS (23, 78), LabBM score (25, 79) and LabPS-score (24) will be assessed at baseline (maximum 4 weeks prior to start of radiotherapy). The scores include the following parameters:

GPS	CRP, albumin
LabBM	CRP, albumin, LDH, hemoglobin, platelets
LabPS	CRP, albumin, LDH, hemoglobin, platelets and ECOG Performance Status

8.4.2. Predictive Biomarkers

Analyses will be performed on MRI and tumor biopsies available from standard diagnostic work-up and follow-up. There will not be performed study-specific procedures, hence, the analyses will be performed in subgroups of patients depending on availability of examinations.

Functional MRI

MRI with functional DW (diffusion-weighted) and/or DCE (dynamic contrast-enhanced) sequences will be analyzed using the Consumption and Supply-based Hypoxia (CSH)-imaging method (27, 66, 80). The hypoxia images will determine the number of voxels located in hypoxic areas within the defined tumor, and the hypoxic fraction (HF) will be assessed.

In addition, available MRI sequences will be explored to obtain tumor information of potential prognostic and/or predictive value.

Molecular Analyses on Tumor Biopsies

Tumor ribonucleic acid (RNA) will be isolated from paraffin-embedded tissue blocks and subjected to global gene expression analysis using the QuantSeq RNA-sequencing method or NanoString technology. These technologies are robust and appropriate for analyses of RNA from paraffin embedded material. The gene expression data will be used to indicate hypoxia by using previously established signatures like the Ragnum signature (67) and the signature by Fjeldbo et al (81, 82). A score will be calculated from the expression levels of the signature genes in the tumor and used in the analyses.

The expression data of all genes in the genome will further be used for explorative analyses to obtain information about signaling pathways that are activated in the tumors. Protein expression will be measured for selected genes by immunohistochemistry based on the paraffin-embedded tissue blocks

PD-L1 expression will be assessed on paraffin-embedded tissue blocks by immunohistochemical staining, and the CPS score will be calculated.

CB-CT

CB-CT acquired during radiotherapy for control of positioning will be explored for evaluation of tumor response and technical aspects of delivered radiotherapy

8.5. Adverse Events (AEs), Serious Adverse Events (SAEs), and Other Safety Reporting

The definitions of AEs and SAEs can be found in Appendix 3. AEs will be reported by the patients, by a caregiver, surrogate, or the patient's legally authorized representative (when appropriate).

The investigators and any qualified designees are responsible for detecting, documenting, and recording events that meet the definition of an AE or SAE and remain responsible for following up all AEs that are serious, considered related to the study intervention, or caused the patient to discontinue the study intervention (see Section 7).

The method of recording, evaluating, and assessing causality of AEs and SAEs and the procedures for completing and transmitting SAE reports are provided in Appendix 3.

8.5.1. Time Period and Frequency of Collecting AE and SAE Information

All SAEs and AEs with CTCAE grade ≥ 3 will be collected from the start of study intervention until the 52 week follow-up visit as specified in the SoA (Section 1.2), and recorded in the eCRF. After the 4 week follow-up visit, only SAEs/AEs with CTCAE grade ≥3 with probable/definite causality with radiotherapy will be reported. For patinets in arm A receiving a second radiotherapy fraction, all SAEs/AEs with CTCAE grade ≥3 will be reported until the 8 week follow-up.

Medical occurrences that begin before the start of study intervention, but after obtaining informed consent will be recorded as current medical history (section 8.1), and not as AEs.

All SAEs will be recorded and reported to the sponsor or designee immediately and under no circumstance should this exceed 24 hours, as indicated in Appendix 3. The investigator will submit any updated SAE data to the sponsor within 24 hours of it being available.

Investigators are not obligated to actively seek information on AEs or SAEs after conclusion of the study participation. However, if the investigator learns of any SAE, including a death, at any time after a patient has been discharged from the study, and he/she considers the event to be reasonably related to the study intervention or study participation, the investigator must promptly notify the sponsor.

8.5.2. Method of Detecting AEs and SAEs

Patients will be instructed to contact their investigator/designated staff at any time after consenting to participate in the study if any symptoms develop. In addition, information on AEs and SAEs will be collected at predefined time points as specified in the SoA (Section 1.2). Care will be taken not to introduce bias when detecting AEs and/or SAEs. Open-ended and non-leading verbal questioning of the patient is the preferred method to inquire about AE occurrences.

8.5.3. Follow-up of AEs and SAEs

After the initial AE/SAE report, the investigator is required to proactively follow each patient at subsequent visits/contacts. All SAEs considered related to the study intervention, or that caused the patient to discontinue the study intervention, will be followed until resolution, stabilization,

the event is otherwise explained, or the patient is lost to follow-up (as defined in Section 7.3. Further information on follow-up procedures is provided in Appendix 10.3.

In the case of a serious AE/SAE, the investigator should initiate the appropriate treatment according to their medical judgement.

Treatment of AEs/SAEs shall not be recorded in the eCRF, only the intensity, causality and outcome see Appendix 10.3.

8.5.4. Regulatory Reporting Requirements for SAEs

- Prompt notification by the investigator to the sponsor of an SAE is essential so that legal obligations and ethical responsibilities towards the safety of patients and the safety of a study intervention under clinical investigation are met.
- The sponsor has a legal responsibility to notify both the local regulatory authority and other regulatory agencies about the safety of a study intervention under clinical investigation. The sponsor will comply with country-specific regulatory requirements relating to safety reporting to the regulatory authority, Institutional Review Boards (IRB)/Independent Ethics Committees (IEC), and investigators.

8.5.5. Pregnancy

- Patients with a positive pregnancy test will not be included due to risk of teratogenic and abortifacient effects of radiotherapy (Section 5.2).
- WOCBP must consent to the use of effective birth control (Appendix 10.4) or abstain from heterosexual activity from randomization and until completed study intervention (Section 5.1)

8.5.6. Disease-Related Events and/or Disease-Related Outcomes Not Qualifying as AEs or SAEs

The following disease-related events are common in patients with metastatic malignant disease and can be serious/life-threatening:

- Event A: hospitalization or death due to disease progression
- Event B: hospitalization for planned investigations or blood/platelet transfusions/fluid transfusions
- Event C: hospitalization for febrile neutropenia if chemotherapy is administered 8-21 days before start of radiotherapy (systemic treatment must be paused one week prior to, during and one week after study intervention)
- Event D: hospitalization for palliative or terminal care

Because these events are typically associated with the disease under study, they will not be reported according to the standard process for expedited reporting of SAEs even though the event may meet the definition of an SAE.

NOTE: However, if either of the following conditions applies, then the event must be recorded and reported as an AE/SAE (instead of a disease-related event):

The event is, in the investigator's opinion, of greater intensity, frequency, or duration than expected for the individual patient.

OR

The investigator considers that there is a reasonable possibility that the event is related to study intervention.

8.6. Pharmacokinetics

Pharmacokinetic parameters will not be evaluated in this study.

8.7. Genetics and/or Pharmacogenomics

Genetics/pharmacogenomics will not be evaluated in this study.

8.8. Biomarkers

Blood test will be collected for assessment of prognostic scores (GPS, LabBM and LabPS), Section 8.4.1. Predictive biomarkers based on medical images and biopsies will be assessed when available, se section 8.4.2.

8.9. Immunogenicity Assessments

Not applicable

8.10. Health Economics OR Medical Resource Utilization and Health Economics

Health economics will be evaluated as indicated in section 8.2.3.

9. Statistical Considerations

9.1. Choice of Non-inferiority Margin

The non-inferiority margin of the difference in the primary endpoint (change in target symptom intensity from baseline after 12 weeks of follow-up) between the two study arms is set to 1 point, which is 50% of the considered minimum clinically important difference based on clinical trials investigating cancer pain (83). The same non-inferiority margin is also currently applied in an ongoing clinical non-inferiority trial (PARASTOP) investigating cancer pain (ClinicalTrials.gov registry number: NCT 05051735). To our judgement, given a different in target symptom relief of less than 1 point, clinicians would prefer radiotherapy with 1-2 fractions of 8 Gy over five fractions of 5 Gy.

9.2. Statistical Hypotheses

The primary hypothesis is that a radiotherapy schedule of 1-2 fractions of 8 Gy provides non-inferior patient-reported symptomatic relief as a schedule of five fractions of 5, assessed 12 weeks after completed radiotherapy (see Section 9.1 for definition of the non-inferiority margin). The lower bound of the 95% confidence interval of the difference in mean symptom intensity of arm A is therefore hypothesized to be above the non-inferiority margin of -1.

If non-inferiority regarding symptom relief, and comparable QoL, toxicities and OS can be proved 12 weeks after the intervention a short-course schedule with 1-2 fractions will be recommend as standard of care. If non-inferiority can be proven only 4 weeks after the intervention, a single fraction schedule will be recommend for patients with a very short life expectancy where palliative radiotherapy is considered reasonable.

9.3. Sample size determination

The sample size determination was based on a one-sided two-sample *t* test. The sample size estimation is based on the following assumptions:

- One-sided T-test with 2,5 % significance level (equivalent to a two-sided 95 % CI)
- Standard deviation (SD) of the primary endpoint equal to 2
- Non-inferiority margin (NIM) of 1

With these assumptions, we will reach a power of 85 % if we enroll 146 patients. Based on the PallRad study, we assume an exclusion from intention to treat (ITT) - population of around 25 % (8, 9), and we will need to randomize a total number of 200 patients.

This number of patients also provide sufficient power to claim non-inferiority on the exploratory endpoints defined in Section 9.1. The exclusion from ITT set is expected to be lower at 4 weeks, increasing the statistical power of conclusions.

9.4. Analysis Sets

The following six analysis sets are defined:

- Intention-to-treat (ITT) set
  - All randomized participants with a signed informed consent form
- Full-analysis (FA) set
  - All randomized, eligible participants with a signed informed consent form who has received at least one radiation fraction
- Per-protocol (PP) set
  - All randomized, eligible participants with a signed informed consent form who has received the allocated radiotherapy and completed the follow-up visits at 4 and 12, and 52 weeks without major protocol deviations
- Modified PP set 1
  - All randomized, eligible participants with a signed informed consent form who has received the allocated radiotherapy and completed the follow-up visits at 4 and 12 weeks without major protocol deviations
- Modified PP set 2
  - All randomized, eligible participants with a signed informed consent form who has received the allocated radiotherapy and completed the follow-up visit at 4 weeks without major protocol deviations
- Safety set
  - All randomized participants exposed to radiotherapy

As this is a non-inferiority study, the main analysis of the primary endpoint will be based on the modified PP set 1 of participants having completed all scheduled follow-up visits at the time of assessment (12 weeks).

The main analyses of the secondary endpoints of physician-reported toxicities (CTCAE) at 4, 12, and 52 weeks will be based on the modified PP set 2, modified PP set 1, and PP set, respectively. Safety analyses of physician-reported toxicities will be based in the safety set.

The main analyses of the secondary endpoint of overall survival will be based on the FA set.

9.5. Statistical analyses

A statistical analysis plan (SAP) will be finalized prior to the database lock, and it will include a more technical and detailed description of the statistical analyses described in this section, which gives a brief summary of the planned statistical analyses of the primary endpoint and secondary endpoints.

9.5.1. General Considerations

A difference between study arms is denoted as statistically significant if the P value is less than the prespecified significance level of 5%. Correspondingly, a confidence level of 95% will be used for the two-sided confidence interval (CI) linear regression models.

9.5.2. Primary Endpoint

Linear regression models will be employed to estimate the difference between the two treatments (change in average target symptom intensity from baseline to the follow up at 12 weeks). We will conclude that 1-2 fractions of 8 Gy (Arm A)is non-inferior to 5 fractions of 5 Gy (Arm B) if the lower bound of the two-sided 95 % confidence interval is above the NIM of -1 (negative values indicate worse outcome for arm A).

Statistical tests for interaction in multivariable linear regression model will be used to assess heterogeneity of radiotherapy treatment effect across different target symptoms.

9.5.3. Secondary Endpoint

Data will be reported as means, 95 % confidence intervals, medians or frequencies as appropriate

<u>CTCAE:</u>

For all; comparison between the two study arms

1. Number of events any grade, any CTCAE category
2. Number of events grade 1-2 vs grade 3-4, any CTCAE-category
3. Number of events any grade, by CTCAE-category
4. Number of events grade 1-2 vs grade 3-4, by CTCAE category
5. Number of patients with event, any CTCAE category
6. Number of patients with events grade 1-2 vs grade 3-4, any CTCAE category
7. Number of patients with events any grade, any CTCAE category
8. Number of patients with grade 1-2 vs grade 3-4 event, by CTCAE category

CTCAE categories: Gastrointestinal, urinary, reproductive system, constitutional, other

Overall survival:

Kaplan Meier- curves compared with the log-rank test and univariable and multivariable Cox proportional-hazards regression analyses

9.5.4. Exploratory endpoints

Analyses of exploratory endpoints will be regarded as hypothesis-generating post-hoc analyses. Continuous endpoints will typically be analyzed by linear regression or median regression, as appropriate. Dichotomous endpoints will typically be analyzed by logistic regression.

9.5.5. Safety Analysis

Safety assessments and reporting of AEs and SAEs and other safety reporting are described in detail in Section 8.3 and Section 8.5, respectively. A Data Monitoring Committee will evaluate safety during the study (Appendix 10.1.4).

The safety set includes all randomized participants exposed to study intervention. General safety evaluations will be based on the occurrence, intensity, and type of AEs (including SAEs) in addition to causality. Safety analyses will be limited to tabulations of AEs/SAEs and subjects with AEs/SAEs.

9.6. Interim Analysis

No interim analysis of efficacy is planned for this study

10. Supporting Documentation and Operational Considerations

10.1. Appendix 1: Regulatory, Ethical, and Study Oversight Considerations

10.1.1. Regulatory and Ethical Considerations

- This study will be conducted in accordance with the protocol and with the following:
  - Consensus ethical principles derived from international guidelines including the Declaration of Helsinki and Council for International Organizations of Medical Sciences (CIOMS) International Ethical Guidelines
  - Applicable ICH Good Clinical Practice (GCP) Guidelines
  - Applicable laws and regulations
- The protocol, protocol amendments, ICF, Investigator Brochure and other relevant documents (e.g., advertisements) must be submitted to an IRB/IEC by the investigator and reviewed and approved by the IRB/IEC before the study is initiated.
- Any amendments to the protocol will require IRB/IEC approval before implementation of changes made to the study design, except for changes necessary to eliminate an immediate hazard to study participants.
- Protocols and any substantial amendments to the protocol will require health authority approval prior to initiation except for changes necessary to eliminate an immediate hazard to study participants.
- The investigator will be responsible for the following:
  - Providing written summaries of the status of the study to the IRB/IEC annually or more frequently in accordance with the requirements, policies, and procedures established by the IRB/IEC
  - Notifying the IRB/IEC of SAEs or other significant safety findings as required by IRB/IEC procedures
  - Providing oversight of the conduct of the study at the site and adherence to requirements of 21 CFR, ICH guidelines, the IRB/IEC, European regulation 536/2014 for clinical studies (if applicable), European Medical Device Regulation 2017/745 for clinical device research (if applicable), and all other applicable local regulations

10.1.2. Informed Consent Process

- The investigator or his/her representative will explain the nature of the study to the participant or their legally authorized representative, and answer all questions regarding the study.

- Participants must be informed that their participation is voluntary. Participants will be required to sign a statement of informed consent that meets the requirements of local regulations, ICH guidelines, and the IRB/IEC or study center.
- The medical record must include a statement that written informed consent was obtained before the participant was enrolled in the study and the date the written consent was obtained. The authorized person obtaining the informed consent must also sign the ICF.
- Participants must be re-consented to the most current version of the ICF(s) during their participation in the study.
- A copy of the ICF(s) must be provided to the participant or their legally authorized representative.

Participants who are rescreened are required to sign a new ICF.

10.1.3. Data Protection

- Participants will be assigned a unique identifier by the sponsor. Any participant records or datasets that are transferred to the sponsor will contain the identifier only; participant names or any information which would make the participant identifiable will not be transferred.
- The participant must be informed that his/her personal study-related data will be used by the sponsor in accordance with local data protection law. The level of disclosure must also be explained to the participant who will be required to give consent for their data to be used as described in the informed consent
- The participant must be informed that his/her medical records may be examined by Clinical Quality Assurance auditors or other authorized personnel appointed by the sponsor, by appropriate IRB/IEC members, and by inspectors from regulatory authorities.

10.1.4. Committees Structure

Data Monitoring Committee (DMC)

A DMC will evaluate safety when 70 included patients have completed the 12 week follow-up, and again when these patients have completed the 52 week follow-up or are registered of study.

Steering Committee

Will oversee the general conduct of the study

10.1.5. Data Quality Assurance

- All participant data relating to the study will be recorded on printed or electronic CRF unless transmitted to the sponsor or designee electronically (e.g., laboratory data). The

investigator is responsible for verifying that data entries are accurate and correct by physically or electronically signing the CRF.

- The investigators must maintain accurate documentation (source data) that supports the information entered in the eCRF.
- Study monitors will perform ongoing source data verification to confirm that data entered into the eCRF by authorized site personnel are accurate, complete and verifiable from source documents; that the safety and rights of participants are being protected; and that the study is being conducted in accordance with the currently approved protocol and any other study agreements, ICH GCP, and all applicable regulatory requirements.
- The investigator must permit study-related monitoring, audits, IRB/IEC review, and regulatory agency inspections and provide direct access to source data documents.
- Monitoring details describing strategy (e.g., risk-based initiatives in operations and quality such as Risk Management and Mitigation Strategies and Analytical Risk-Based Monitoring), methods, responsibilities and requirements, including handling of noncompliance issues and monitoring techniques (central, remote, or on-site monitoring) are provided in the Monitoring Plan.
- The sponsor or designee is responsible for the data management of this study including quality checking of the data.
- The sponsor assumes accountability for actions delegated to other individuals (e.g., Contract Research Organizations).
- Records and documents, including signed ICFs, pertaining to the conduct of this study must be retained by the investigator for 15 years after study completion unless local regulations or institutional policies require a longer retention period. No records may be destroyed during the retention period without the written approval of the sponsor. No records may be transferred to another location or party without written notification to the sponsor.

10.1.6. Source Documents

- Source documents provide evidence for the existence of the participant and substantiate the integrity of the data collected. Source documents are filed at the investigator’s site.
- Data reported on the CRF or entered in the eCRF that are transcribed from source documents must be consistent with the source documents or the discrepancies must be explained. The investigator may need to request previous medical records or transfer records, depending on the study. Also, current medical records must be available.
- Definition of what constitutes source data can be found in the Investigator Site File.

- The investigator must maintain accurate documentation (source data) that supports the information entered in the CRF.
- Study monitors will perform ongoing source data verification to confirm that data entered into the CRF by authorized site personnel are accurate, complete, and verifiable from source documents; that the safety and rights of participants are being protected; and that the study is being conducted in accordance with the currently approved protocol and any other study agreements, ICH GCP, and all applicable regulatory requirements.

10.1.7. Study and Site Start and Closure

First Act of Recruitment

The study start date is the date on which the clinical study will be open for recruitment of participants.

The first act of recruitment is the first site open and will be the study start date.

Study/Site Termination

The sponsor or designee reserves the right to close the study site or terminate the study at any time for any reason at the sole discretion of the sponsor. Study sites will be closed upon study completion. A study site is considered closed when all required documents and study supplies have been collected and a study-site closure visit has been performed.

The investigator may initiate study-site closure at any time, provided there is reasonable cause and sufficient notice is given in advance of the intended termination.

Reasons for the early closure of a study site by the sponsor or investigator may include but are not limited to:

For study termination:

- Discontinuation of further study intervention development

For site termination:

- Failure of the investigator to comply with the protocol, the requirements of the IRB/IEC or local health authorities, the sponsor's procedures, or GCP guidelines
- Inadequate or no recruitment (evaluated after a reasonable amount of time) of participants by the investigator
- Total number of participants included earlier than expected

If the study is prematurely terminated or suspended, the sponsor shall promptly inform the investigators, the IECs/IRBs, the regulatory authorities, and any contract research organization(s) used in the study of the reason for termination or suspension, as specified by the applicable

regulatory requirements. The investigator shall promptly inform the participant and should assure appropriate participant therapy and/or follow-up

10.1.8. Publication Policy

- The results of this study will be published in international, peer review journals and will be presented at scientific meetings. If this is foreseen, the investigator agrees to submit all manuscripts or abstracts to the sponsor before submission. This allows the sponsor to protect proprietary information and to provide comments.
- The sponsor will comply with the requirements for publication of study results. In accordance with standard editorial and ethical practice, the sponsor will generally support publication of multicenter studies only in their entirety and not as individual site data. In this case, a coordinating investigator will be designated by mutual agreement.
- Authorship will be determined by mutual agreement and in line with International Committee of Medical Journal Editors authorship requirements.

10.2. Appendix 2: Clinical Laboratory Tests

See section 8.3.3 and 8.3.5.

10.3. Appendix 3: AEs and SAEs: Definitions and Procedures for Recording, Evaluating, Follow-up, and Reporting

10.3.1. Definition of AE

**AE Definition**
• An AE is any untoward medical occurrence in a clinical study participant, temporally associated with the use of study intervention, whether or not considered related to the study intervention.<br>• NOTE: An AE can therefore be any unfavorable and unintended sign (including an abnormal laboratory finding), symptom, or disease (new or exacerbated) temporally associated with the use of study intervention.

**Events Meeting the AE Definition**
• Any abnormal laboratory test results (haematology, clinical chemistry, or urinalysis) or other safety assessments (e.g., ECG, radiological scans, vital signs measurements), including those that worsen from baseline, considered clinically significant in the medical and scientific judgment of the investigator (i.e., not related to progression of underlying disease).<br>• Exacerbation of a chronic or intermittent pre-existing condition including either an increase in frequency and/or intensity of the condition.<br>• New conditions detected or diagnosed after study intervention administration even though it may have been present before the start of the study.<br>• Signs, symptoms, or the clinical sequelae of a suspected intervention- intervention interaction.<br>• Signs, symptoms, or the clinical sequelae of a suspected overdose of either study intervention or a concomitant medication. Overdose per se will not be reported as an AE/SAE unless it is an intentional overdose taken with possible suicidal/self-harming intent. Such overdoses should be reported regardless of sequelae.<br>• The signs, symptoms, and/or clinical sequelae resulting from lack of efficacy will be reported as AE or SAE if they fulfil the definition of an AE or SAE. “Lack of efficacy” or “failure of expected pharmacological action” also constitutes an AE or SAE.

**Events NOT Meeting the AE Definition**

- Any clinically significant abnormal laboratory findings or other abnormal safety assessments which are associated with the underlying disease, unless judged by the investigator to be more severe than expected for the participant's condition.
- The disease/disorder being studied or expected progression, signs, or symptoms of the disease/disorder being studied, unless more severe than expected for the participant's condition.
- Medical or surgical procedure (e.g., endoscopy, appendectomy): the condition that leads to the procedure is the AE.
- Situations in which an untoward medical occurrence did not occur (social and/or convenience admission to a hospital).
- Anticipated day-to-day fluctuations of pre-existing disease(s) or condition(s) present or detected at the start of the study that do not worsen.

10.3.2. Definition of SAE

An SAE is defined as any serious adverse event that, at any dose:

a. Results in death

- All deaths that occur during the study intervention, or within the protocol-defined follow-up period must be reported as follows:
  - Death clearly resulting from disease progression should be recorded in the eCRF, and should not be recorded as a SAE
  - Where death is not due (or not clearly due) to disease progression, the AE must be reported as a SAE
  - The report should contain a comment regarding the co-involvement of progressive disease, if appropriate, and should assign main and contributory causes of death
  - Deaths with an unknown cause should always be reported as a SAE

b. Is life-threatening

The term 'life-threatening' in the definition of 'serious' refers to an event in which the participant was at risk of death at the time of the event. It does not refer to an event, which hypothetically might have caused death, if it were more severe.

c. Requires inpatient hospitalization or prolongation of existing hospitalization

- In general, hospitalization signifies that the participant has been admitted (usually involving at least an overnight stay) at the hospital or emergency ward for observation

and/or treatment that would not have been appropriate in the physician's office or outpatient setting. Complications that occur during hospitalization are AEs. If a complication prolongs hospitalization or fulfills any other serious criteria, the event is serious. When in doubt as to whether "hospitalization" occurred or was necessary, the AE should be considered serious.<br>• Any elective hospitalization or admission to hospice or nursing home that was planned prior to randomization will not meet SAE-criteria.<br>• Hospitalization (including admission to hospice or nursing home) due to worsening of the underlying condition will only be recorded as a SAE if medically judged there was an unexpected worsening due to study intervention
**d. Results in persistent or significant disability/incapacity**<br>• The term disability means a substantial disruption of a person's ability to conduct normal life functions.<br>• This definition is not intended to include experiences of relatively minor medical significance such as uncomplicated headache, nausea, vomiting, diarrhea, influenza, and accidental trauma (e.g., sprained ankle) which may interfere with or prevent everyday life functions but do not constitute a substantial disruption.
**e. Is a congenital anomaly/birth defect**
**f. Other situations:**<br>• Medical or scientific judgment should be exercised by the investigator in deciding whether SAE reporting is appropriate in other situations such as significant medical events that may jeopardize the participant or may require medical or surgical intervention to prevent one of the other outcomes listed in the above definition. These events should usually be considered serious.<br>o Examples of such events include invasive or malignant cancers, intensive treatment for allergic bronchospasm, blood dyscrasias, convulsions or development of intervention dependency or intervention abuse.

10.3.3. Recording and Follow-Up of AE and/or SAE

**AE and SAE Recording**
• When an AE/SAE occurs, it is the responsibility of the investigator to review all documentation (e.g., hospital progress notes, laboratory reports, and diagnostics reports) related to the event.

- Radiotherapy treatment in both study arms are currently being used in clinical practice. Study participants will have numerous AEs related to their disease and nearby systemic treatment. All AEs ≥CTCAE grade 3 and relevant information will be reported
- It is **not** acceptable for the investigator to send photocopies of the participant's medical records to sponsor or designee in lieu of completion of the AE/SAE CRF page.
- There may be instances when copies of medical records for certain cases are requested by sponsor. In this case, all participant identifiers, with the exception of the participant number, will be redacted on the copies of the medical records before submission to sponsor.
- The investigator will attempt to establish a diagnosis of the event based on signs, symptoms, and/or other clinical information. Whenever possible, the diagnosis (not the individual signs/symptoms) will be documented as the AE/SAE.

Assessment of Intensity

The investigator will make an assessment of intensity for each AE and SAE reported during the study using the National Cancer Institute Common Terminology Criteria for Adverse Events (CTCAE) v 5.1). If an AEs or an SAEs are not covered by CTCAE, it will be assigned to 1 of the following categories:

- Mild: An event that is easily tolerated by the participant, causing minimal discomfort and not interfering with everyday activities.
- Moderate: An event that causes sufficient discomfort to interfere with normal everyday activities.
- Severe: An event that prevents normal everyday activities. An AE that is assessed as severe should not be confused with an SAE. Severe is a category utilized for rating the intensity of an event; and both AEs and SAEs can be assessed as severe.

  An event is defined as 'serious' when it meets at least 1 of the predefined outcomes as described in the definition of an SAE, NOT when it is rated as severe.

Assessment of Causality

- The investigator is obligated to assess the relationship between study intervention and each occurrence of each AE/SAE.
- A "reasonable possibility" of a relationship conveys that there are facts, evidence, and/or arguments to suggest a causal relationship, rather than a relationship cannot be ruled out.

- The investigator will use clinical judgment to determine the relationship.
- Alternative causes, such as underlying disease(s), concomitant therapy, and other risk factors, as well as the temporal relationship of the event to study intervention administration will be considered and investigated.
- For each AE/SAE, the investigator **must** document in the medical notes that he/she has reviewed the AE/SAE and has provided an assessment of causality.
- There may be situations in which an SAE has occurred and the investigator has minimal information to include in the initial report to sponsor. However, it is very important that the investigator always make an assessment of causality for every event before the initial transmission of the SAE data to sponsor.
- The investigator may change his/her opinion of causality in light of follow-up information and send an SAE follow-up report with the updated causality assessment.
- The causality assessment is one of the criteria used when determining regulatory reporting requirements.

**Follow-up of AEs and SAEs**
• The investigator is obligated to perform or arrange for the conduct of supplemental measurements and/or evaluations as medically indicated or as requested by medical monitor or sponsors designee to elucidate the nature and/or causality of the AE or SAE as fully as possible. This may include additional laboratory tests or investigations, histopathological examinations, or consultation with other health care professionals.<br>• New or updated information will be recorded in the originally submitted documents.<br>• The investigator will submit any updated SAE data to sponsor within 24 hours of receipt of the information.

10.3.4. Reporting of SAEs

**SAE Reporting to sponsor or sponsors designee via an Electronic Data Collection Tool**
• The primary mechanism for reporting an SAE will be the electronic data collection tool.<br>• If the electronic system is unavailable, then the site will use the paper SAE data collection tool (see next section) to report the event within 24 hours.<br>• The site will enter the SAE data into the electronic system as soon as it becomes available.

- After the study is completed at a given site, the electronic data collection tool will be taken off-line to prevent the entry of new data or changes to existing data.
- If a site receives a report of a new SAE from a study participant or receives updated data on a previously reported SAE after the electronic data collection tool has been taken off-line, then the site can report this information on a paper SAE form (see next section) or to the sponsor by telephone.
- Contacts for SAE reporting can be found in the Procedures Manual.

SAE Reporting to sponsor or sponsors designee via Paper Data Collection Tool

- If the eCRF is not operable, facsimile transmission of the SAE paper data collection tool is the preferred method to transmit this information to the sponsor or sponsors designee
- In rare circumstances and in the absence of facsimile equipment, notification by telephone is acceptable with a copy of the SAE data collection tool sent by overnight mail or courier service.
- Initial notification via telephone does not replace the need for the investigator to complete and sign the SAE data collection tool within the designated reporting time frames.
- Contacts for SAE reporting can be found in the Procedures Manual.

10.4. Appendix 4: Contraceptive and Barrier Guidance

WOCBP should be willing to use a highly effective methods for birth control, be surgically sterile, or abstain from heterosexual activity from randomization until completed study intervention as detailed below:

- Combined (estrogen and progestogen containing) hormonal contraception associated with inhibition of ovulation (oral, intravaginal or transdermal)
- Progestogen-only hormonal contraception associated with inhibition of ovulation (oral, injectable or implantable)
- Intrauterine device (IUD)
- Intrauterine hormone-releasing system (IUS)
- Bilateral tubal occlusion
- Vasectomized partner
- Heterosexual abstinence

10.5. Appendix 5: Abbreviations

The following is a list of abbreviations that may be used in the protocol

AE	Adverse Event
AR	Adverse Reaction
CI	Confidence Interval
eCRF	Electronic Case Report Form
CTA	Clinical Trial Authorization
CTC	Clinical Trial Coordinator
CTCAE	Common Terminology Criteria for Adverse Events
CTU	Clinical Trials Unit
DMC	Data Monitoring Committee
EORTC	European Organization for Research and Treatment of Cancer
GCP	Good Clinical Practice
Gy	Grey
IB	Investigators Brochure
ICF	Informed Consent Form
ICH	International Conference of Harmonization
IEC	Independent Ethics Committee
IMP	Investigational Medicinal Product
IRB	Institutional Review Board
ISF	Investigator Site File

NRS	Numerical Rating Scale
NCI	National Cancer Institute
NIM	Non-Inferiority Margin
PGIC	Patient Global Impression of Change
PS	Performance status
REC	Research Ethics Committee
SAE	Serious Adverse Event
SAR	Serious Adverse Reaction
SD	Standard Deviation
SoA	Schedule of Activities
SOP	Standard Operating Procedure
SUSAR	Suspected Unexpected Serious Adverse Reaction
TMF	Trial Master File
TMG	Trial Management Group
TSC	Trial Steering Committee
UAR	Unexpected Adverse Reaction
WOCBP	Women of ChildBearing Potential

10.6. Appendix 6: Common Terminology Criteria for Adverse Events V 5.0

The descriptions and grading scales found in the NCI Common Terminology Criteria for Adverse Events (CTCAE) version 5.0 will be utilized for adverse event reporting, and is available at Common Terminology Criteria for Adverse Events (CTCAE) | Protocol Development | CTEP (cancer.gov)

11. References

1. Lawler M, Davies L, Oberst S, Oliver K, Eggermont A, Schmutz A, et al. European Groundshot-addressing Europe's cancer research challenges: a Lancet Oncology Commission. Lancet Oncol. 2023;24(1):e11-e56.

2. Cameron MG, Kersten C, Guren MG, Fosså SD, Vistad I. Palliative pelvic radiotherapy of symptomatic incurable prostate cancer - a systematic review. Radiother Oncol. 2014;110(1):55-60.

3. Cameron MG, Kersten C, Vistad I, Fosså S, Guren MG. Palliative pelvic radiotherapy of symptomatic incurable rectal cancer - a systematic review. Acta Oncol. 2014;53(2):164-73.

4. Fabian A, Domschikowski J, Letsch A, Schmalz C, Freitag-Wolf S, Dunst J, Krug D. Clinical endpoints in trials of palliative radiotherapy: A systematic meta-research analysis. Radiother Oncol. 2022;174:123-31.

5. Wilson MK, Karakasis K, Oza AM. Outcomes and endpoints in trials of cancer treatment: the past, present, and future. Lancet Oncol. 2015;16(1):e32-42.

6. Lievens Y, Dunscombe P, Defourny N, Gasparotto C, Borras JM, Grau C. HERO (Health Economics in Radiation Oncology): a pan-European project on radiotherapy resources and needs. Clin Oncol (R Coll Radiol). 2015;27(2):115-24.

7. Skliarenko J, Barnes EA. Palliative pelvic radiotherapy for gynaecologic cancer. Journal of Radiation Oncology. 2012;1(3):239-44.

8. Cameron MG, Kersten C, Vistad I, van Helvoirt R, Weyde K, Undseth C, et al. Palliative pelvic radiotherapy for symptomatic rectal cancer - a prospective multicenter study. Acta Oncol. 2016;55(12):1400-7.

9. Cameron MG, Kersten C, Vistad I, van Helvoirt R, Weyde K, Undseth C, et al. Palliative pelvic radiotherapy for symptomatic incurable prostate cancer – A prospective multicenter study. Radiother Oncol. 2015;115(3):314-20.

10. Farina E, Macchia G, Siepe G, Zamagni A, Buwenge M, Scirocco E, et al. Palliative Short-course Radiotherapy in Advanced Pelvic Cancer: A Phase II Study (SHARON Project). Anticancer Res. 2019;39(8):4237-42.

11. Dhiman S, Rai B, Vamsi K, Gowda S, Anand D, Miriyala R, et al. Hypofractionated Two Week Short-Course Radiotherapy vs. Monthly Single Fraction Palliative Pelvic Radiation in Advanced Gynecologic Cancers. International Journal of Radiation Oncology*Biology*Physics. 2022;114(3, Supplement):S16-S7.

12. Lutz ST, Jones J, Chow E. Role of radiation therapy in palliative care of the patient with cancer. J Clin Oncol. 2014;32(26):2913-9.

13. Duchesne GM, Bolger JJ, Griffiths GO, Trevor Roberts J, Graham JD, Hoskin PJ, et al. A randomized trial of hypofractionated schedules of palliative radiotherapy in the management of bladder carcinoma: results of medical research council trial BA09. Int J Radiat Oncol Biol Phys. 2000;47(2):379-88.

14. Raby SEM, Hoskin P, Choudhury A. The role of palliative radiotherapy in bladder cancer: a narrative review. Ann Palliat Med. 2020;9(6):4294-9.

15. Kombathula SH, Cree A, Joshi PV, Akturk N, Barraclough LH, Haslett K, et al. Palliative radiotherapy in cancers of female genital tract: Outcomes and prognostic factors. Radiother Oncol. 2022;175:42-6.

16. Picardi V, Deodato F, Guido A, Giaccherini L, Macchia G, Frazzoni L, et al. Palliative Short-Course Radiation Therapy in Rectal Cancer: A Phase 2 Study. Int J Radiat Oncol Biol Phys. 2016;95(4):1184-90.

17. Kaasa S, Brenne E, Lund JA, Fayers P, Falkmer U, Holmberg M, et al. Prospective randomised multicenter trial on single fraction radiotherapy (8 Gy x 1) versus multiple fractions (3 Gy x 10) in the treatment of painful bone metastases. Radiother Oncol. 2006;79(3):278-84.

18. Chow E, Harris K, Fan G, Tsao M, Sze WM. Palliative radiotherapy trials for bone metastases: a systematic review. J Clin Oncol. 2007;25(11):1423-36.

19. Chow E, van der Linden YM, Roos D, Hartsell WF, Hoskin P, Wu JS, et al. Single versus multiple fractions of repeat radiation for painful bone metastases: a randomised, controlled, non-inferiority trial. Lancet Oncol. 2014;15(2):164-71.

20. Sundstrøm S, Bremnes R, Aasebø U, Aamdal S, Hatlevoll R, Brunsvig P, et al. Hypofractionated palliative radiotherapy (17 Gy per two fractions) in advanced non-small-cell lung carcinoma is comparable to standard fractionation for symptom control and survival: a national phase III trial. J Clin Oncol. 2004;22(5):801-10.

21. Stevens R, Macbeth F, Toy E, Coles B, Lester JF. Palliative radiotherapy regimens for patients with thoracic symptoms from non-small cell lung cancer. Cochrane Database Syst Rev. 2015;1(1):Cd002143.

22. Jones JA, Lutz ST, Chow E, Johnstone PA. Palliative radiotherapy at the end of life: a critical review. CA Cancer J Clin. 2014;64(5):296-310.

23. Nieder C, Mannsåker B, Dalhaug A, Pawinski A, Haukland E. The Glasgow prognostic score: Useful information when prescribing palliative radiotherapy. Mol Clin Oncol. 2017;6(6):811-6.

24. Nieder C, Yobuta R, Mannsåker B. Expansion of the LabBM Score: Is the LabPS the Best Tool Predicting Survival in Patients With Brain Metastases? Am J Clin Oncol. 2021;44(2):53-7.

25. Nieder C, Dalhaug A, Haukland E. The LabBM score is an excellent survival prediction tool in patients undergoing palliative radiotherapy. Rep Pract Oncol Radiother. 2021;26(5):740-6.

26. Salberg UB, Skingen VE, Fjeldbo CS, Hompland T, Ragnum HB, Vlatkovic L, et al. A prognostic hypoxia gene signature with low heterogeneity within the dominant tumour lesion in prostate cancer patients. Br J Cancer. 2022;127(2):321-8.

27. Hompland T, Hole KH, Ragnum HB, Aarnes EK, Vlatkovic L, Lie AK, et al. Combined MR Imaging of Oxygen Consumption and Supply Reveals Tumor Hypoxia and Aggressiveness in Prostate Cancer Patients. Cancer Res. 2018;78(16):4774-85.

28. Yang L, West CM. Hypoxia gene expression signatures as predictive biomarkers for personalising radiotherapy. Br J Radiol. 2019;92(1093):20180036.

29. Horsman MR, Overgaard J. The impact of hypoxia and its modification of the outcome of radiotherapy. J Radiat Res. 2016;57 Suppl 1(Suppl 1):i90-i8.

30. Wu CT, Chen WC, Chang YH, Lin WY, Chen MF. The role of PD-L1 in the radiation response and clinical outcome for bladder cancer. Sci Rep. 2016;6:19740.

31. Camilleri-Brennan J, Steele RJ. The impact of recurrent rectal cancer on quality of life. Eur J Surg Oncol. 2001;27(4):349-53.

32. Cancer in Norway: Norwegian Cancer Registry; 2021 [Available from: https://www.kreftregisteret.no.

33. Guren MG, Kørner H, Pfeffer F, Myklebust T, Eriksen MT, Edna TH, et al. Nationwide improvement of rectal cancer treatment outcomes in Norway, 1993-2010. Acta Oncol. 2015;54(10):1714-22.

34. Braendengen M, Tveit KM, Berglund A, Birkemeyer E, Frykholm G, Påhlman L, et al. Randomized phase III study comparing preoperative radiotherapy with chemoradiotherapy in nonresectable rectal cancer. J Clin Oncol. 2008;26(22):3687-94.

35. van Gijn W, Marijnen CA, Nagtegaal ID, Kranenbarg EM, Putter H, Wiggers T, et al. Preoperative radiotherapy combined with total mesorectal excision for resectable rectal cancer: 12-year follow-up of the multicentre, randomised controlled TME trial. Lancet Oncol. 2011;12(6):575-82.

36. Benitez Majano S, Di Girolamo C, Rachet B, Maringe C, Guren MG, Glimelius B, et al. Surgical treatment and survival from colorectal cancer in Denmark, England, Norway, and Sweden: a population-based study. Lancet Oncol. 2019;20(1):74-87.

37. Islami F, Ferlay J, Lortet-Tieulent J, Bray F, Jemal A. International trends in anal cancer incidence rates. Int J Epidemiol. 2017;46(3):924-38.

38. Guren MG, Aagnes B, Nygård M, Dahl O, Møller B. Rising Incidence and Improved Survival of Anal Squamous Cell Carcinoma in Norway, 1987-2016. Clin Colorectal Cancer. 2019;18(1):e96-e103.

39. Gilbert DC, Serup-Hansen E, Linnemann D, Høgdall E, Bailey C, Summers J, et al. Tumour-infiltrating lymphocyte scores effectively stratify outcomes over and above p16 post chemo-radiotherapy in anal cancer. Br J Cancer. 2016;114(2):134-7.

40. Serup-Hansen E, Linnemann D, Skovrider-Ruminski W, Høgdall E, Geertsen PF, Havsteen H. Human papillomavirus genotyping and p16 expression as prognostic factors for patients with American Joint Committee on Cancer stages I to III carcinoma of the anal canal. J Clin Oncol. 2014;32(17):1812-7.

41. Rao S, Guren MG, Khan K, Brown G, Renehan AG, Steigen SE, et al. Anal cancer: ESMO Clinical Practice Guidelines for diagnosis, treatment and follow-up(☆). Ann Oncol. 2021;32(9):1087-100.

42. Parker C, Castro E, Fizazi K, Heidenreich A, Ost P, Procopio G, et al. Prostate cancer: ESMO Clinical Practice Guidelines for diagnosis, treatment and follow-up. Ann Oncol. 2020;31(9):1119-34.

43. Powles T, Bellmunt J, Comperat E, De Santis M, Huddart R, Loriot Y, et al. Bladder cancer: ESMO Clinical Practice Guideline for diagnosis, treatment and follow-up. Ann Oncol. 2022;33(3):244-58.

44. Landoni F, Maneo A, Colombo A, Placa F, Milani R, Perego P, et al. Randomised study of radical surgery versus radiotherapy for stage Ib-IIa cervical cancer. Lancet. 1997;350(9077):535-40.

45. Oonk MHM, Planchamp F, Baldwin P, Bidzinski M, Brännström M, Landoni F, et al. European Society of Gynaecological Oncology Guidelines for the Management of Patients With Vulvar Cancer. Int J Gynecol Cancer. 2017;27(4):832-7.

46. Lu KH, Broaddus RR. Endometrial Cancer. N Engl J Med. 2020;383(21):2053-64.

47. Raja FA, Chopra N, Ledermann JA. Optimal first-line treatment in ovarian cancer. Ann Oncol. 2012;23 Suppl 10:x118-27.

48. Johnson N, Bryant A, Miles T, Hogberg T, Cornes P. Adjuvant chemotherapy for endometrial cancer after hysterectomy. Cochrane Database Syst Rev. 2011;2011(10):Cd003175.

49. Miller DS, Filiaci VL, Mannel RS, Cohn DE, Matsumoto T, Tewari KS, et al. Carboplatin and Paclitaxel for Advanced Endometrial Cancer: Final Overall Survival and Adverse Event Analysis of a Phase III Trial (NRG Oncology/GOG0209). J Clin Oncol. 2020;38(33):3841-50.

50. Colombo N, Sessa C, du Bois A, Ledermann J, McCluggage WG, McNeish I, et al. ESMO-ESGO consensus conference recommendations on ovarian cancer: pathology and molecular biology, early and advanced stages, borderline tumours and recurrent disease†. Ann Oncol. 2019;30(5):672-705.

51. Rogers L, Siu SS, Luesley D, Bryant A, Dickinson HO. Radiotherapy and chemoradiation after surgery for early cervical cancer. Cochrane Database Syst Rev. 2012;5(5):Cd007583.

52. Pötter R, Tanderup K, Kirisits C, de Leeuw A, Kirchheiner K, Nout R, et al. The EMBRACE II study: The outcome and prospect of two decades of evolution within the GEC-ESTRO GYN working group and the EMBRACE studies. Clin Transl Radiat Oncol. 2018;9:48-60.

53. Vergote I, Tropé CG, Amant F, Kristensen GB, Ehlen T, Johnson N, et al. Neoadjuvant chemotherapy or primary surgery in stage IIIC or IV ovarian cancer. N Engl J Med. 2010;363(10):943-53.

54. Joiner M, van der Kogel A. Basic Clinical Radiobiology: Routledge; 2018.

55. Withers HR. Biologic basis for altered fractionation schemes. Cancer. 1985;55(9 Suppl):2086-95.

56. van Leeuwen CM, Oei AL, Crezee J, Bel A, Franken NAP, Stalpers LJA, Kok HP. The alfa and beta of tumours: a review of parameters of the linear-quadratic model, derived from clinical radiotherapy studies. Radiat Oncol. 2018;13(1):96.

57. Stevens R, Macbeth F, Toy E, Coles B, Lester JF. Palliative radiotherapy regimens for patients with thoracic symptoms from non-small cell lung cancer. Cochrane Database of Systematic Reviews. 2015(1).

58. Chow E, Harris K, Fan G, Tsao M, Sze WM. Palliative Radiotherapy Trials for Bone Metastases: A Systematic Review. Journal of Clinical Oncology. 2007;25(11):1423-36.

59. Laugsand TS, Kaasa S, Romundstad P, Johannesen TB, Lund J. Radiotherapy for bone metastases: practice in Norway 1997-2007. A national registry-based study. Acta Oncol. 2013;52(6):1129-36.

60. Radu C, Berglund A, Påhlman L, Glimelius B. Short-course preoperative radiotherapy with delayed surgery in rectal cancer - a retrospective study. Radiother Oncol. 2008;87(3):343-9.

61. van Dijk TH, Tamas K, Beukema JC, Beets GL, Gelderblom AJ, de Jong KP, et al. Evaluation of short-course radiotherapy followed by neoadjuvant bevacizumab, capecitabine, and oxaliplatin and subsequent radical surgical treatment in primary stage IV rectal cancer. Ann Oncol. 2013;24(7):1762-9.

62. Nieder C, Andratschke NH, Grosu AL. Personalized radiotherapy of brain metastases: survival prediction by means of dichotomized or differentiated blood test results? Front Oncol. 2023;13:1156161.

63. Hanahan D, Weinberg RA. Hallmarks of cancer: the next generation. Cell. 2011;144(5):646-74.

64. Thiruthaneeswaran N, Bibby BAS, Yang L, Hoskin PJ, Bristow RG, Choudhury A, West C. Lost in application: Measuring hypoxia for radiotherapy optimisation. Eur J Cancer. 2021;148:260-76.

65. Vaupel P, Höckel M, Mayer A. Detection and characterization of tumor hypoxia using pO2 histography. Antioxid Redox Signal. 2007;9(8):1221-35.

66. Skipar K, Hompland T, Lund KV, Løndalen A, Malinen E, Kristensen GB, et al. Risk of recurrence after chemoradiotherapy identified by multimodal MRI and 18F-FDG-PET/CT in locally advanced cervical cancer. Radiother Oncol. 2022;176:17-24.

67. Ragnum HB, Vlatkovic L, Lie AK, Axcrona K, Julin CH, Frikstad KM, et al. The tumour hypoxia marker pimonidazole reflects a transcriptional programme associated with aggressive prostate cancer. Br J Cancer. 2015;112(2):382-90.

68. Dermani FK, Samadi P, Rahmani G, Kohlan AK, Najafi R. PD-1/PD-L1 immune checkpoint: Potential target for cancer therapy. J Cell Physiol. 2019;234(2):1313-25.

69. Mercier A, Conan-Charlet V, Quintin-Roué I, Doucet L, Marcorelles P, Uguen A. Reproducibility in PD-L1 Immunohistochemistry Quantification through the Tumor Proportion Score and the Combined Positive Score: Could Dual Immunostaining Help Pathologists? Cancers (Basel). 2023;15(10).

70. De Martino M, Daviaud C, Vanpouille-Box C. Radiotherapy: An immune response modifier for immuno-oncology. Semin Immunol. 2021;52:101474.

71. Tyc-Szczepaniak D, Kepka L, Pietrzak L, Olszyna-Serementa M, Bujko K, Wyrwicz L, et al. Palliative radiotherapy and chemotherapy instead of surgery in symptomatic rectal cancer with synchronous unresectable metastases: a phase II study†. Annals of Oncology. 2013;24(11):2829-34.

72. Rijkmans EC, van Triest B, Nout RA, Kerkhof EM, Buijsen J, Rozema T, et al. Evaluation of clinical and endoscopic toxicity after external beam radiotherapy and endorectal brachytherapy in elderly patients with rectal cancer treated in the HERBERT study. Radiotherapy and oncology : journal of the European Society for Therapeutic Radiology and Oncology. 2018;126(3):417-23.

73. Halle JS, Rosenman JG, Varia MA, Fowler WC, Walton LA, Currie JL. 1000 CGY single dose palliation for advanced carcinoma of the cervix or endometrium. International Journal of Radiation Oncology*Biology*Physics. 1986;12(11):1947-50.

74. Onsrud M, Hagen B, Strickert T. 10-Gy single-fraction pelvic irradiation for palliation and life prolongation in patients with cancer of the cervix and corpus uteri. Gynecologic oncology. 2001;82(1):167-71.

75. Yan J, Milosevic M, Fyles A, Manchul L, Kelly V, Levin W. A hypofractionated radiotherapy regimen (0-7-21) for advanced gynaecological cancer patients. Clinical oncology. 2011;23(7):476-81.

76. Spanos WJ, Jr., Clery M, Perez CA, Grigsby PW, Doggett RL, Poulter CA, Steinfeld AD. Late effect of multiple daily fraction palliation schedule for advanced pelvic malignancies (RTOG 8502). Int J Radiat Oncol Biol Phys. 1994;29(5):961-7.

77. Alderson P, Chalmers I. Survey of claims of no effect in abstracts of Cochrane reviews. Bmj. 2003;326(7387):475.

78. Anshushaug M, Gynnild MA, Kaasa S, Kvikstad A, Grønberg BH. Characterization of patients receiving palliative chemo- and radiotherapy during end of life at a regional cancer center in Norway. Acta Oncol. 2015;54(3):395-402.

79. Berghoff AS, Wolpert F, Holland-Letz T, Koller R, Widhalm G, Gatterbauer B, et al. Combining standard clinical blood values for improving survival prediction in patients with newly diagnosed brain metastases-development and validation of the LabBM score. Neuro Oncol. 2017;19(9):1255-62.

80. Hillestad T, Hompland T, Fjeldbo CS, Skingen VE, Salberg UB, Aarnes EK, et al. MRI Distinguishes Tumor Hypoxia Levels of Different Prognostic and Biological Significance in Cervical Cancer. Cancer Res. 2020;80(18):3993-4003.

81. Fjeldbo CS, Julin CH, Lando M, Forsberg MF, Aarnes EK, Alsner J, et al. Integrative Analysis of DCE-MRI and Gene Expression Profiles in Construction of a Gene Classifier for Assessment of Hypoxia-Related Risk of Chemoradiotherapy Failure in Cervical Cancer. Clin Cancer Res. 2016;22(16):4067-76.

82. Halle C, Andersen E, Lando M, Aarnes EK, Hasvold G, Holden M, et al. Hypoxia-induced gene expression in chemoradioresistant cervical cancer revealed by dynamic contrast-enhanced MRI. Cancer Res. 2012;72(20):5285-95.

83. Caraceni A, Hanks G, Kaasa S, Bennett MI, Brunelli C, Cherny N, et al. Use of opioid analgesics in the treatment of cancer pain: evidence-based recommendations from the EAPC. Lancet Oncol. 2012;13(2):e58-68.
