# Supplementary material for: Palliative radiotherapy in symptomatic pelvic soft tissue tumors (PallSoft)– protocol for a national, randomized, non-inferiority study
Source: BMC Cancer. 2025 Jul 1;25:1051. doi: 10.1186/s12885-025-14424-1 (PMC12211963; doi:10.1186/s12885-025-14424-1)
Supplement: Supplementary file 3 — Supplementary Material 3 [file 12885_2025_14424_MOESM3_ESM.docx]

**Palliative radiotherapy in symptomatic pelvic Soft tissue tumors (PallSoft) – protocol for a national, randomized, non-inferiority study**

**Authors:**

Kjersti Skipar, MD^a^*^,^ Maren S. Ørvik, MD^a^, Christoph Evers, MD, PhD^b^, Lise Balteskard, MD, PhD^c^, Christian Ekanger, MD^d^, Liv Ellen Giske, MD^e^ , Kjersti Ødegaard, MD^f^, Elin H. Østrem, MD^g^, Cecilie S. Nordstrand, MD^h^, Hanne Tøndel, MD, PhD^i^, Carsten Nieder, MD, prof ^j,k^ , Marianne G. Guren^l,m^, MD, Prof, Stein Kaasa, MD, prof ^l,m^, Harald B. Ragnum MD, PhD^a^

**Affiliations**

^a^ Department of Oncology, Telemark Hospital Trust, Skien, Norway

^b^ Center for Cancer Treatment, Hospital of Southern Norway, Kristiansand, Norway

^c^ Department of Oncology, University Hospital of North Norway, Tromsø, Norway

^d^ Department of Oncology and Medical Physics, Haukeland University Hospital, Bergen, Norway

^e^ Department of Oncology, Gjøvik Hospital, Innlandet Hospital Trust, Gjøvik, Norway

^f^ Department of Oncology, Stavanger University Hospital, Stavanger, Norway

^g^ Section of oncology, Drammen Hospital, Vestre Viken Hospital Trust, Drammen, Norway

^h^ Department of Oncology, Ålesund Hospital, Møre and Romsdal Hospital Trust, Ålesund, Norway

^i^ Cancer Clinic, St. Olavs Hospital Trust, Trondheim University Hospital, Trondheim, Norway

^j^ Department of Oncology and Palliative Medicine, Nordland Hospital, Bodø, Norway

^k^ Department of Clinical Medicine, Faculty of Health Sciences, University of Tromsø, Tromsø, Norway

^l^ Department of Oncology, Oslo University Hospital, Oslo, Norway

^m^ Institute of Clinical Medicine, University of Oslo, Oslo, Norway

^n^ European Palliative Care Research Centre (PRC)

***Corresponding author**

Kjersti Skipar

Email: [kjeski@sthf.no](mailto:kjeski@sthf.no)

Postal Address: Telemark Hospital Trust, Ulefossvegen 55, 3710 Skien, Norway

**Trial sponsor:** Telemark Hospital Trust

**Participating hospitals:**

Telemark Hospital Trust, Skien, Norway

Hospital of Southern Norway, Kristiansand, Norway

University Hospital of North Norway, Tromsø, Norway

Haukeland University Hospital, Bergen, Norway

Innlandet Hospital Trust, Gjøvik, Norway

Stavanger University Hospital, Stavanger, Norway

Vestre Viken Hospital Trust, Drammen, Norway

Møre and Romsdal Hospital Trust, Ålesund, Norway

St. Olavs Hospital Trust, Trondheim University Hospital, Trondheim, Norway

Nordland Hospital, Bodø, Norway

Oslo University Hospital, Oslo, Norway

**Protocol version 2.4, May 2025**
